# Supplementary material for: Neoadjuvant Afatinib for stage III EGFR-mutant non-small cell lung cancer: a phase II study
Source: Nat Commun. 2023 Aug 3;14:4655. doi: 10.1038/s41467-023-40349-z (PMC10400609; doi:10.1038/s41467-023-40349-z)
Supplement: Supplementary file 1 — Supplementary Information [file 41467_2023_40349_MOESM1_ESM.pdf]

Supplementary Table 1. Baseline information in different mutant status and operable status

	Mutant Subtype No. (%)			Diagnostic Operable Assessment No. (%)		
**Variables**	**Ex19Del**	**L858R**	***p***	**Resectable**	**Potentially Resectable**	***p***
**No. of Patients**	28 (63.6)	16 (36.4)		22 (46.8)	25 (53.2)	
**Age, years (range)**	60.0 (33-78)	61.6 (39-76)	0.772*	61.4 (45-76)	59.9 (33-78)	0.657*
**Gender**			0.864			0.920
Male	13 (46.4)	7 (43.8)		10 (45.5)	11 (44.0)	
Female	15 (53.6)	9 (56.2)		12 (54.5)	14 (56.0)	
**ECOG Performance Status**			0.598			0.145
0	18 (64.3)	9 (56.2)		16 (72.7)	13 (52.0)	
1	10 (35.7)	7 (43.8)		6 (27.3)	12 (48.0)	
**Smoking Status**			0.634			0.918
Ever	7 (25.0)	3 (18.8)		5 (22.7)	6 (24.0)	
Never	21 (75.0)	13 (81.2)		17 (77.3)	19 (76.0)	
**MTD at Diagnosis, mm (range)**	43.0 (20-93)	43.0 (18-76)	0.887	39.7 (21-64)	46.3 (18-93)	0.192
**N Stage at Diagnosis**			0.638			/
1	4 (14.3)	1 (6.3)		4 (18.2)	1 (4.0)	
2	19 (67.9)	10 (62.5)		18 (81.8)	14 (56.0)	
3	5 (17.8)	5 (31.2)		0 (0.0)	10 (40.0)	
**cTNM Stage at Diagnosis**			0.490			/
IIIA	17 (60.7)	8 (50.0)		22 (100.0)	4 (16.0)	
IIIB	9 (32.1)	7 (43.8)		0 (0.0)	18 (72.0)	
IIIC	2 (7.2)	1 (6.2)		0 (0.0)	3 (12.0)	

*: Continuous variable was compared using independent samples t-test. The other categorical variables were compared using chi-squares test or Fisher's exact

test.

MTD: maximum tumor dimension.

Supplementary Table 2. The characteristics of patients with uncommon *EGFR*-mutant subtypes (n =3)

ID	ECOG	Smoking	Mutation	T (mm)	N Stage	cTNM	Cycle	c-Regression			Surgery	yp-Regression	
								RECIST	Tumor	Node		Residual Tumor	Node
014	0	Never	S768I	53	2	IIIB	2	SD	17%	SD	R0	95%	N0
044	1	Never	G719X	42	2	IIIB	4	PR	33%	PR	Without	/	/
048	0	40 PY	G719X	45	2	IIIA	2	PR	31%	CR	R0	35%	N2

CR: complete response; PR: partial response; R0: complete resection; SD: stable disease.

Supplementary Table 3. Correlation between EFS and the parameters should be re-evaluated after neoadjuvant Afatinib treatment (n = 46)

**Variables**	**HR**	**95% CI**	***p***
**Reduction of SLD (%)**	0.954	0.924-0.985	0.004
**Pathological Response (%)**	0.974	0.937-1.014	0.200
**Surgical Treatment**	1.743	0.364-8.333	0.487

The time-dependent Cox model was used for comparison.

CI: confidence interval; HR: hazard ratio; SLD: sum of lesion diameter.

Supplementary Table 4. Comparison of the efficacy of neoadjuvant treatment in Ex19Del and L858R mutant patients (n = 44)

**Variables**	**Ex19Del**<br>No. (%)	**L858R**<br>No. (%)	***p***
**No. of Patients**	28 (63.6)	16 (36.4)	
**Operable Evaluation at Diagnosis**			0.098
Resectable	16 (57.1)	5 (31.3)	
Potentially Resectable	12 (42.9)	11 (68.7)	
**Duration of NAT, cycle (range)**	2.6 (1-9)	2.9 (2-6)	0.938
**Surgical Treatment**			0.852
Performed	20 (71.4)	11 (68.8)	
Non-Performed	8 (28.6)	5 (31.2)	
**Tumor Response**			0.235
PR	18 (64.3)	13 (81.3)	
Non-PR	10 (35.7)	3 (18.7)	
**ORR (%)**	64.3	81.3	
**DCR (%)**	89.3	100.0	
**MTD Regression, % (range)**	35.3 (-28%-78%)	38.9 (14%-76%)	0.046
**ypTNM**			0.641
0/I/II	11 (55.0)	7 (63.6)	
III	9 (45.0)	4 (36.4)	
**Type of Surgery** #			0.436
Thoracotomy	8 (40.0)	6 (54.5)	
VATS	12 (60.0)	5 (45.5)	
**Surgical Resection** #			0.818
Lobectomy	17 (85.0)	9 (81.8)	
Bi-Lobectomy	3 (15.0)	2 (18.2)	
**Resection** #			0.639
R0	17 (85.0)	10 (90.9)	
R1+R2	3 (15.0)	1 (9.1)	
**Residual Tumor Cell, % (range)** #	57.5 (0%-90%)	55.5 (10%-90%)	0.875*
**Residual Tumor Cell** #			0.808
≤ 60%	10 (50.0)	6 (54.5)	
> 60%	10 (50.0)	5 (45.5)	
**Pathologic Regression** #			0.933
pCR	1 (5.0)	0 (0.0)	
MPR	2 (10.0)	1 (9.1)	
Non-MPR	18 (90.0)	10 (90.9)	

#: Patients received neoadjuvant therapy followed by surgery (n = 33).

*: Continuous variable was compared using independent samples t-test. The other categorical variables were compared using chi-squares test or Fisher's exact test.

DCR: disease control rate; MPR: major pathological response; MTD: maximum tumor

dimension; NAT: neoadjuvant Afatinib treatment; ORR: objective response rate; pCR: pathological complete response; PR: partial response; R0: complete resection; R1+R2: uncomplete resection; VATS: video-assisted thoracic surgery.

Supplementary Table 5. Comparison of the efficacy of neoadjuvant treatment in resectable and potentially resectable patients (n = 47)

**Variables**	**Resectable**<br>No. (%)	**Potentially Resectable**<br>No. (%)	***p***
**No. of Patients**	22 (46.8)	25 (53.2)	
**EGFR Mutation**			0.226
Ex19Del	16 (72.7)	12 (48.0)	
L858R	5 (22.7)	11 (44.0)	
Uncommon	1 (4.6)	2 (8.0)	
**Duration of NAT, cycle (range)**	2.2 (1-4)	3.2 (1-9)	0.016
**Surgical Treatment**			
Performed	20 (90.9)	13 (52.0)	
Non-Performed	2 (9.1)	12 (48.0)	
**Tumor Response**			0.355
PR	14 (63.6)	19 (76.0)	
Non-PR	8 (36.4)	6 (24.0)	
**ORR (%)**	63.6	76.0	
**DCR (%)**	95.5	92.0	
**MTD Regression, % (range)**	33.3 (-26-78)	38.5 (-28-76)	0.459
**ypTNM** #			0.710
0/I/II	11 (55.0)	8 (61.5)	
III	9 (45.0)	5 (38.5)	
**Type of Surgery** #			0.226
Thoracotomy	8 (40.0)	8 (61.5)	
VATS	12 (60.0)	5 (38.5)	
**Surgical Resection** #			0.306
Lobectomy	18 (90.0)	10 (76.9)	
Bi-Lobectomy	2 (10.0)	3 (23.1)	
**Resection** #			/
R0	20 (100.0)	9 (69.2)	
R1+R2	0 (0.0)	4 (30.8)	
**Residual Tumor Cell, % (range)** #	57.8 (0-90)	56.5 (10-95)	0.914*
**Residual Tumor Cell** #			0.828
≤ 60%	10 (50.0)	7 (53.8)	
> 60%	10 (50.0)	6 (46.2)	
**Pathologic Regression** #			0.821
pCR	1 (5.0)	0 (0.0)	
MPR	2 (10.0)	1 (7.7)	
Non-MPR	18 (90.0)	12 (92.3)	

#: Patients received neoadjuvant therapy followed by surgery (n = 33).

*: Continuous variable was compared using independent samples t-test. The other categorical variables were compared using chi-squares test or Fisher's exact test.

DCR: disease control rate; MPR: major pathological response; MTD: maximum tumor dimension; NAT: neoadjuvant Afatinib treatment; ORR: objective response rate; pCR: pathological complete response; PR: partial response; R0: complete resection; R1+R2: uncomplete resection; VATS: video-assisted thoracic surgery.

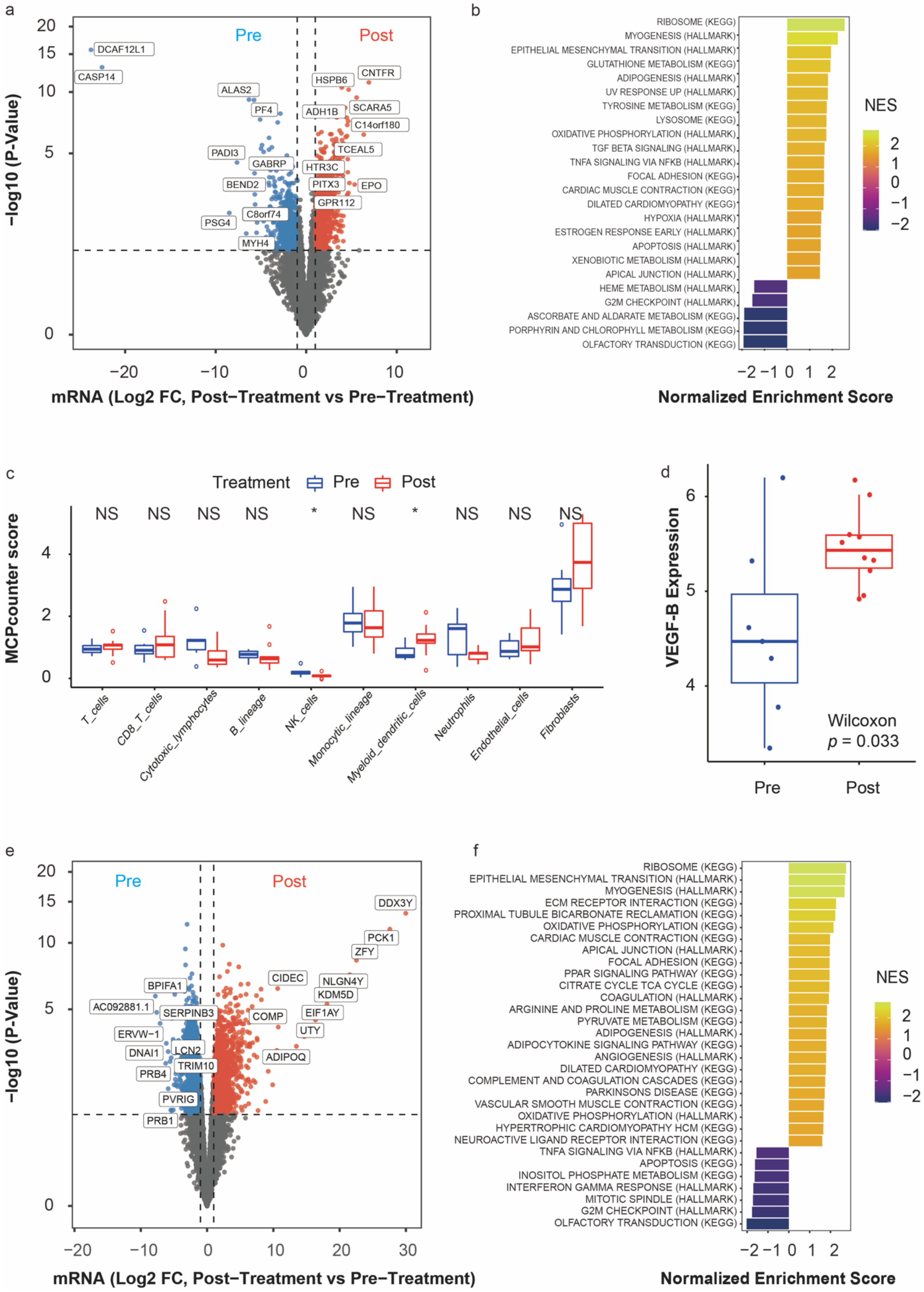

Supplementary Figure 1: The changes of transcriptomic features and immune cell infiltration

between baseline and post-treatment tumor and LN samples in non-responders. (a) Differential expression between baseline (n = 7) and post-treatment tumor samples (n = 10). (b) GSEA analysis between baseline and post-treatment tumor samples. (c) Immune cells differences between baseline (n = 7) and post-treatment tumor samples (n = 10), *: $p < 0.05$; NS: $p \geq 0.05$; $p = 0.67$, $p = 0.54$, $p = 0.11$, $p = 0.36$, $p = 0.033$, $p = 0.81$, $p = 0.043$, $p = 0.11$, $p = 0.42$, $p = 0.16$ from left to right. (d) Increasing VEGF-B expression in tumor samples post-treatment (n = 10) compared with baseline (n = 7). (e) Differential expression between baseline (n = 4) and post-treatment LN (n = 4) samples. (f) GSEA analysis between baseline (n = 4) and post-treatment LN (n = 4) samples. Centers, boxes, whiskers, and dots indicate medians, quantiles, minima/maxima, and outliers, respectively in c-d. Two-sided Wilcoxon rank sum test was used for comparison in c-d. Source data are provided as a Source Data file. NS: no significance.

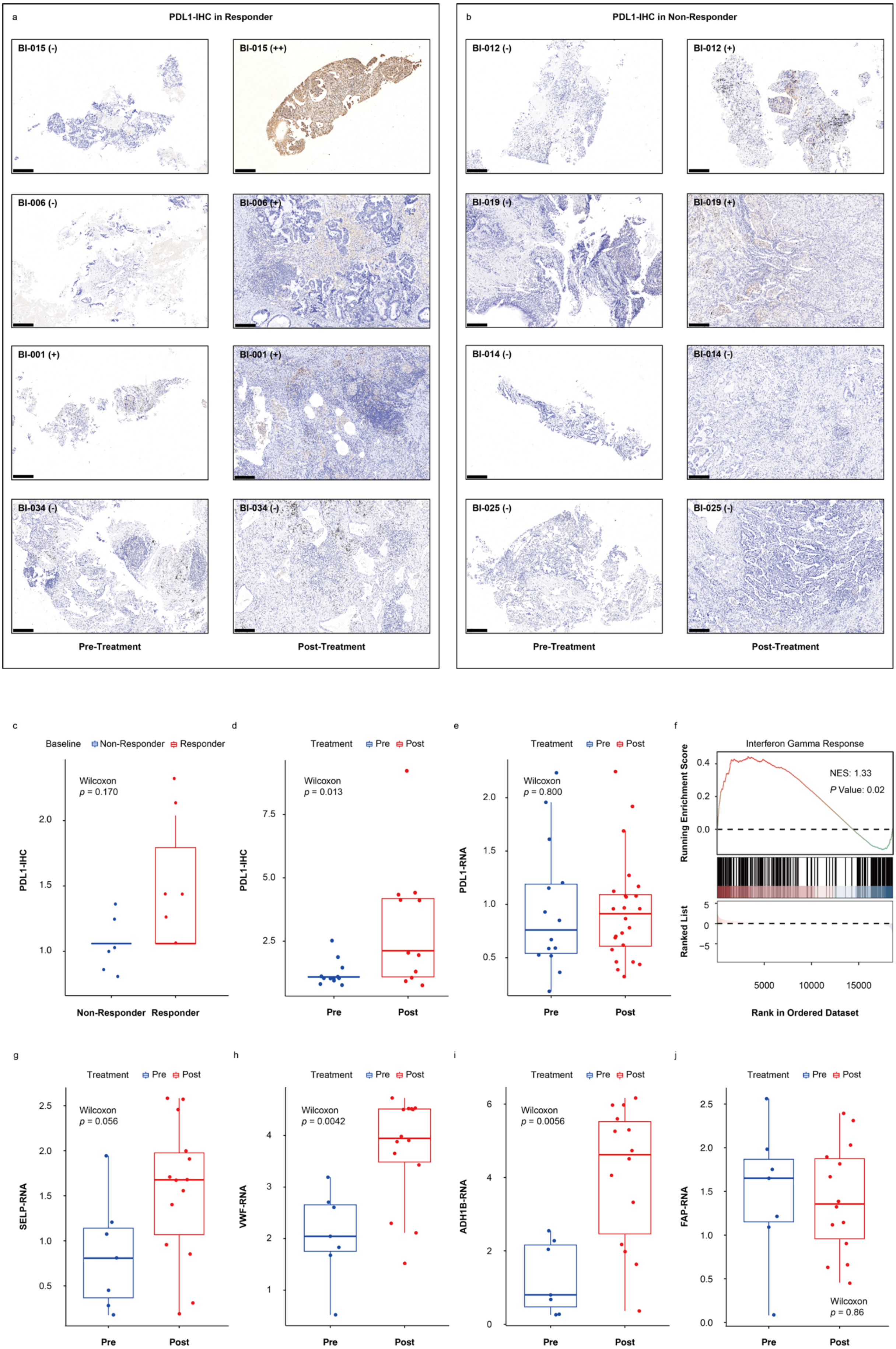
a
PDL1-IHC in Responder
BI-015 (-)
BI-015 (++)
BI-006 (-)
BI-006 (+)
BI-001 (+)
BI-001 (+)
BI-034 (-)
BI-034 (-)
Pre-Treatment
Post-Treatment
b
PDL1-IHC in Non-Responder
BI-012 (-)
BI-012 (+)
BI-019 (-)
BI-019 (+)
BI-014 (-)
BI-014 (-)
BI-025 (-)
BI-025 (-)
Pre-Treatment
Post-Treatment
c
Baseline Non-Responder Responder
Wilcoxon
p = 0.170
PDL1-IHC
Non-Responder Responder
d
Treatment Pre Post
Wilcoxon
p = 0.013
PDL1-IHC
Pre
Post
e
Treatment Pre Post
Wilcoxon
p = 0.800
PDL1-RNA
Pre
Post
f
Interferon Gamma Response
NES: 1.33
P Value: 0.02
Running Enrichment Score
Ranked List
Rank in Ordered Dataset
g
Treatment Pre Post
Wilcoxon
p = 0.056
SELP-RNA
Pre
Post
h
Treatment Pre Post
Wilcoxon
p = 0.0042
VWF-RNA
Pre
Post
i
Treatment Pre Post
Wilcoxon
p = 0.0056
ADH1B-RNA
Pre
Post
j
Treatment Pre Post
Wilcoxon
p = 0.86
FAP-RNA
Pre
Post

Supplementary Figure 2: The changes of expression of PD-L1 and enrichment of IFNG pathway between baseline and post-treatment tumor with different responses. (a) The IHC images (10X) of PD-L1 in pre-treatment and post-treatment tumor samples from responders. (b) The IHC images (10X) of PD-L1 in pre-treatment and post-treatment tumor samples from non-responders. (c) High PD-L1 IHC expression tendency in baseline responder tumor samples (n = 6) than non-responder tumor samples (n = 6). (d) Significantly high PD-L1 IHC expression in post-treatment tumor samples (n = 11) than baseline tumor samples (n = 12). (e) High PD-L1 RNA-seq expression tendency in post-treatment tumor samples (n = 24) compared with baseline (n = 14). (f) Significant enrichment of IFNG pathway in post-treatment responder tumor samples (n = 14). High SELP (g), VWF (h) and ADHIB (i) but low FAP (j) RNA-seq expression in post-treatment responder tumor samples (n = 14) compared with baseline (n = 7). Scare bar, 200 μm for 10X images. Centers, boxes, whiskers, and dots indicate medians, quantiles, minima/maxima, and outliers, respectively in c-e and g-j. Two-sided Wilcoxon rank sum test was used for comparison in c-e and g-j. Source data are provided as a Source Data file. IHC: immunohistochemistry.

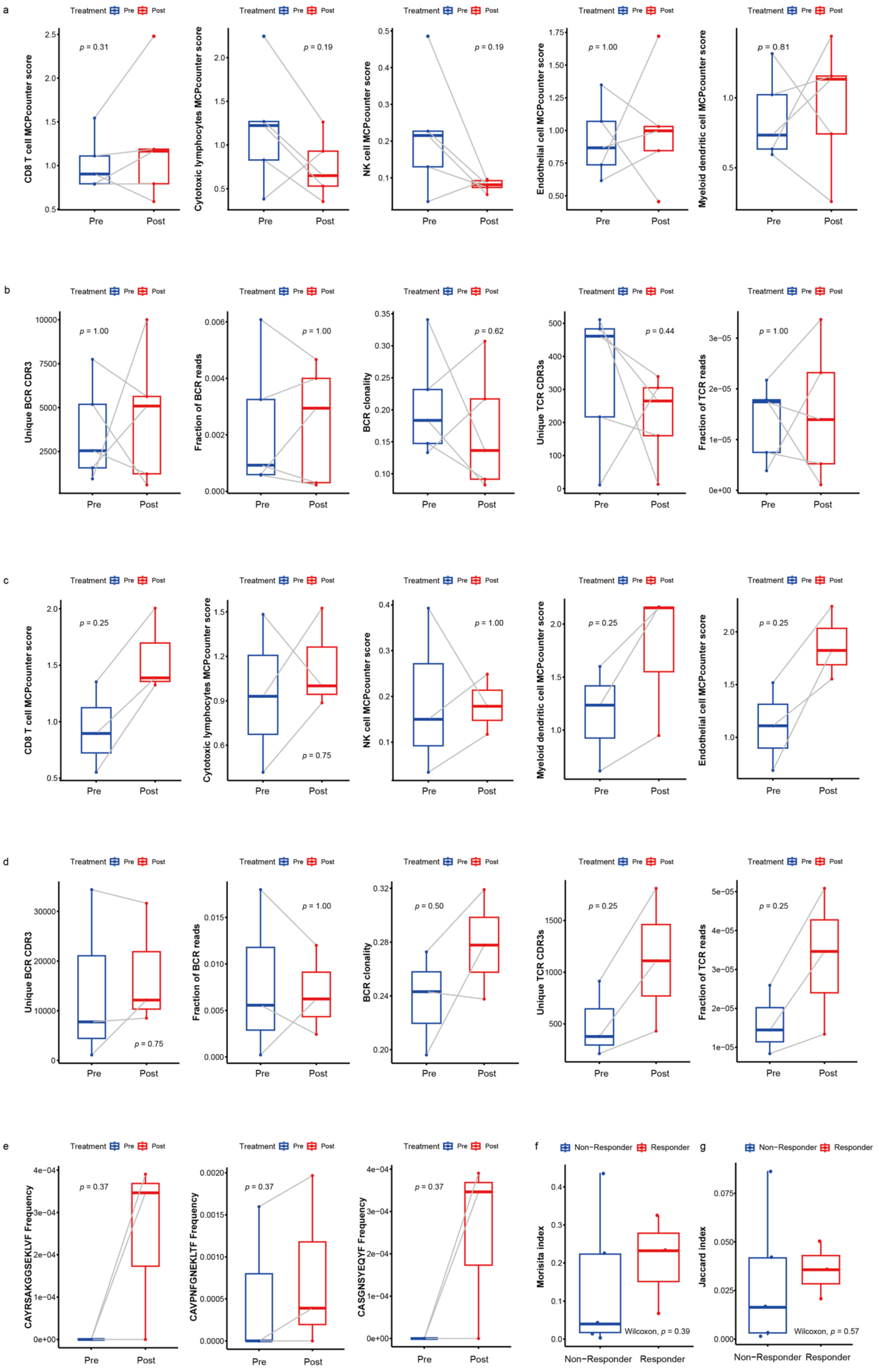
a
Treatment Pre Post
CD8 T cell MCPcounter score
p = 0.31
Cytotoxic lymphocytes MCPcounter score
p = 0.19
NK cell MCPcounter score
p = 0.19
Endothelial cell MCPcounter score
p = 1.00
Myeloid dendritic cell MCPcounter score
p = 0.81
Pre Post
b
Unique BCR CDR3
p = 1.00
Fraction of BCR reads
p = 1.00
BCR clonality
p = 0.62
Unique TCR CDR3s
p = 0.44
Fraction of TCR reads
p = 1.00
c
CD8 T cell MCPcounter score
p = 0.25
Cytotoxic lymphocytes MCPcounter score
p = 0.75
NK cell MCPcounter score
p = 1.00
Myeloid dendritic cell MCPcounter score
p = 0.25
Endothelial cell MCPcounter score
p = 0.25
d
Unique BCR CDR3
p = 0.75
Fraction of BCR reads
p = 1.00
BCR clonality
p = 0.50
Unique TCR CDR3s
p = 0.25
Fraction of TCR reads
p = 0.25
e
CAYRSAKGGSEKLVF Frequency
p = 0.37
CAVPNFGNEKLTF Frequency
p = 0.37
CASGNSYEQYF Frequency
p = 0.37
f
Non−Responder Responder
Morisita index
Wilcoxon, p = 0.39
g
Non−Responder Responder
Jaccard index
Wilcoxon, p = 0.57

Supplementary Figure 3: The changes of immune cell infiltration and immune repertoire between baseline and post-treatment tumor in matched samples. (a) Changes in immune cells from pre- to post-treatment tumor samples in non-responders (n = 5). (b) Changes in immune repertoire from pre- to post-treatment tumor samples in non-responders (n = 5). (c) Changes in immune cells from pre- to post-treatment tumor samples in responders (n = 3). (d) Changes in immune repertoire from pre- to post-treatment tumor samples in responders (n = 3). (e) Treatment-associated clones from pre- to post-treatment tumor samples in responders (n = 3). High jaccard (f) and morisita (g) index in responders (n = 3) compared with non-resonders (n = 5). Centers, boxes, whiskers, and dots indicate medians, quantiles, minima/maxima, and outliers, respectively. Two-sided Wilcoxon rank sum test was used for comparison. Source data are provided as a Source Data file.

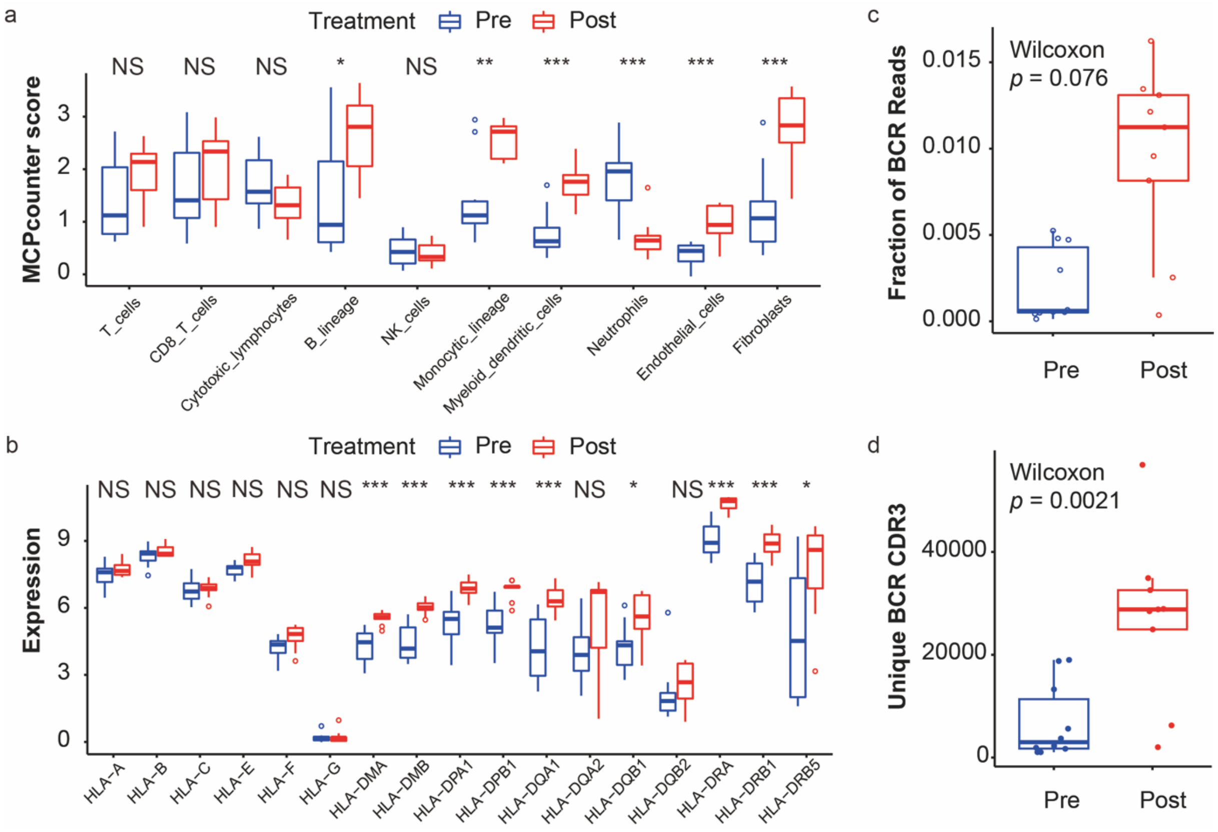
a
Treatment Pre Post
NS NS NS * NS ** *** *** *** ***
MCPcounter score
0
1
2
3
T_cells
CD8_T_cells
Cytotoxic_lymphocytes
B_lineage
NK_cells
Monocytic_lineage
Myeloid_dendritic_cells
Neutrophils
Endothelial_cells
Fibroblasts
b
Treatment Pre Post
NS NS NS NS NS NS *** *** *** *** *** NS * NS *** *** *
Expression
0
3
6
9
HLA−A
HLA−B
HLA−C
HLA−E
HLA−F
HLA−G
HLA−DMA
HLA−DMB
HLA−DPA1
HLA−DPB1
HLA−DQA1
HLA−DQA2
HLA−DQB1
HLA−DQB2
HLA−DRA
HLA−DRB1
HLA−DRB5
c
Wilcoxon
p = 0.076
Fraction of BCR Reads
0.000
0.005
0.010
0.015
Pre
Post
d
Wilcoxon
p = 0.0021
Unique BCR CDR3
0
20000
40000
Pre
Post

Supplementary Figure 4: The changes of immune cell infiltration between baseline and post-treatment LN samples in responders. (a) Increasing B cells in post-treatment LN samples (n = 9) compared with baseline (n = 10), $p = 0.11$, $p = 0.36$, $p = 0.4$, $p = 0.043$, $p = 0.84$, $p = 0.0041$, $p = 0.00065$, $p = 0.00065$, $p = 0.00097$, $p = 0.00065$ from left to right. (b) Increasing MHC-II family members in post-treatment LN samples (n = 9) compared with baseline (n = 10), $p = 0.66$, $p = 0.4$, $p = 0.6$, $p = 0.079$, $p = 0.053$, $p = 0.66$, $p = 0.00015$, $p = 0.00015$ $p = 0.00026$, $p = 0.00041$, $p = 0.00041$, $p = 0.053$, $p = 0.01$, $p = 0.32$, $p = 0.00015$, $p = 0.00065$, $p = 0.022$ from left to right. (c) Increasing fractions of BCR reads and (d) Unique BCR CDR3 in post-treatment LN samples (n = 9) compared with baseline (n = 10). Centers, boxes, whiskers, and dots indicate medians, quantiles, minima/maxima, and outliers, respectively. Two-sided Wilcoxon rank sum test was used for comparison. *: $p < 0.05$; **: $p < 0.01$; ***: $p < 0.001$; NS: $p \geq 0.05$ in a-b. Source data are provided as a Source Data file. BCR: B-cell receptor; NS: no significance.

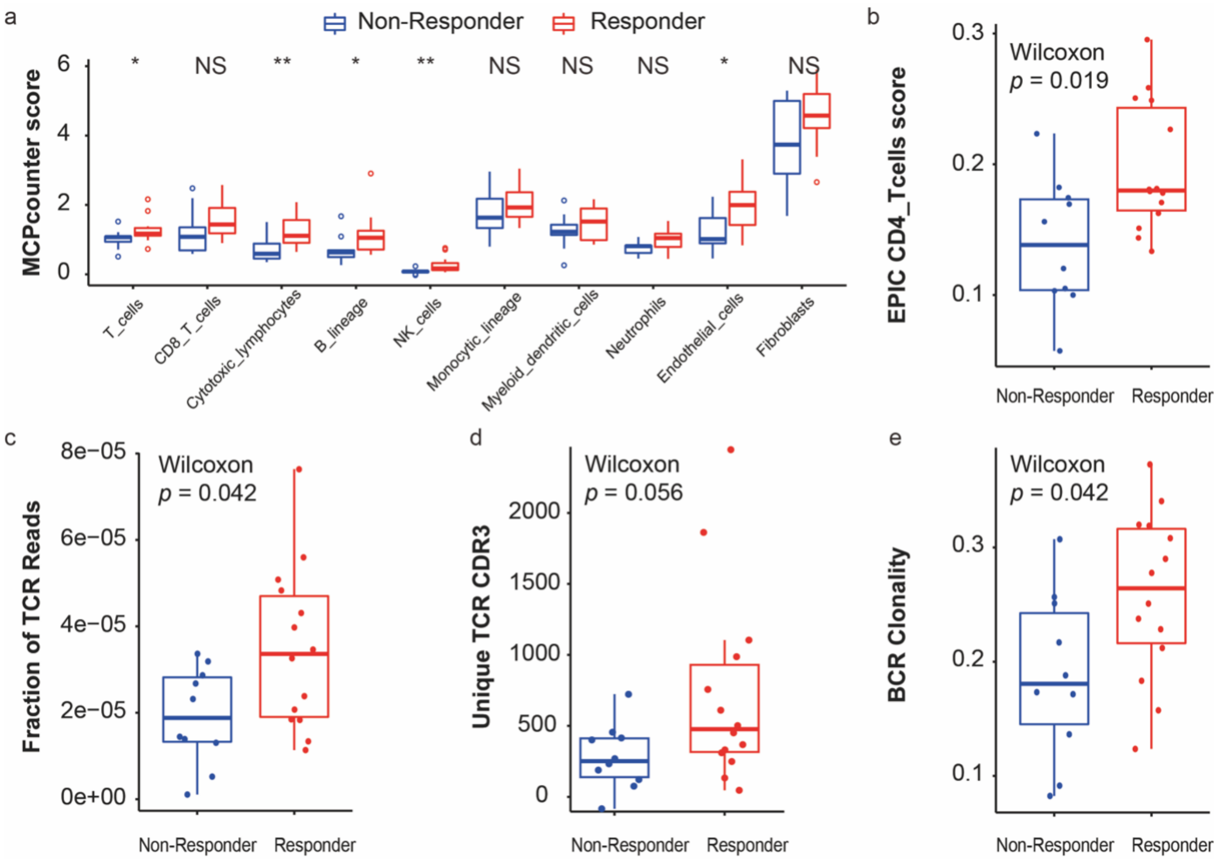

Supplementary Figure 5: Immune environment differences in post-treatment tumor samples with different responses. (a) Increasing T cells and B cells infiltration in post-treatment responder tumors (n = 14) compared with non-responder (n = 10) via MCPcounter, *: $p < 0.05$; **: p < 0.01; NS: $p \geq 0.05$; $p = 0.019$, $p = 0.096$, $p = 0.0059$, $p = 0.042$, $p = 0.0019$, $p = 0.19$, $p = 0.31$, $p = 0.074$, $p = 0.019$, $p = 0.15$ from left to right. (b) Increasing CD4+ T cells infiltration in post-treatment responder tumors (n = 14) compared with non-responder (n = 10) via EPIC. (c) Increasing fractions of TCR reads, (d) Unique TCR CDR3 and (e) BCR clonality in post-treatment responder tumors (n = 14) compared with non-responder (n = 10). Centers, boxes, whiskers, and dots indicate medians, quantiles, minima/maxima, and outliers, respectively. Two-sided Wilcoxon rank sum test was used for comparison. Source data are provided as a Source Data file. BCR: B-cell receptor; NS: no significance; TCR: T-cell receptor

Neoadjuvant Afatinib Therapy for Stage III EGFR Mutation-Positive Lung Adenocarcinoma: A Single-Arm, Open-Label, Phase II Clinical Trial

Investigator: Professor Zhang Peng

Institution: Shanghai Pulmonary Hospital of Tongji University

Study Period: Oct. 2019 to Dec. 2025

Version: 2.1

Version Date: 2019-12-27

Confidentiality Statement

The information contained in this document, especially the unpublished data, is owned by the Sponsor of this study and is provided to you as confidential since you are considered as an investigator, potential investigator or consultant for review by you, your research team and the independent ethics committee/institutional review committee. It must be made clear that such confidential information is not allowed to be disclosed to others without the written approval from the Sponsor, except that it is used to obtain the Informed Consent Form from patients who will possibly take the study drugs.

Ptotocol Summary

Title:	Neoadjuvant Afatinib Therapy for Potentially Resectable Stage III EGFR Mutation-Positive Lung Adenocarcinoma: A Single-Arm, Open-Label, Phase II Clinical Trial
Version No.:	2.1
Research Institution:	Shanghai Pulmonary Hospital of Tongji University
Organization in Charge:	Shanghai Pulmonary Hospital of Tongji University
Principal Investigator:	Professor Zhang Peng
Participants:	Patients with potentially resectable stage III EGFR mutation-positive lung adenocarcinoma
Objectives:	To evaluate the efficacy and safety of neoadjuvant Afatinib in the treatment of stage III EGFR mutation-positive lung adenocarcinoma.
Study Design:	Single-arm, open-Label, phase II clinical trial
Sample Size:	47 in total
Test Drug:	Afatinib (trade name: Giotrif)
Dosage and Mode of Administration	40mg ,Tablet, PO,QD
Trial Content:	① Neoadjuvant treatment stage: the enrolled patients take afatinib at a dosage of 40mg per day, 8-16 weeks in total; and receive CT scan re-examination within 3th post-therapy week. (To get an accurate ORR, the patients should receive CT scan several days later instead of doing it immediately after stopping taking afatinib)（RECIST1.1）.<br>② Surgical treatment stage: the patients who respond to Afatinib treatment (CR+PR) and the patients who do not respond to Afatinib therapy but could still undergo surgery (SD and PD) will receive radical lung lobectomy+systematic lymph node dissection three weeks (22 days) later. ((After neoadjuvant therapy the tissue will be congestive and edematous due to inflammation interaction. If there isn’t an interval, it will bring a high risk of perioperative complications. According to our clinical experience, a three-

week interval is preferred)).

③ Adjuvant treatment stage: The CR, PR and SD patients who have been treated surgically will take Afatinib at a dosage of 40mg per day for 1 year. The SD and PD patients who could not receive operation and the PD patients who have received operation will be transferred into medical oncology or/and radiation oncology and receive comprehensive therapy (chemotherapy or/and radiotherapy, the regimen is designed by oncologist and radiologist).

Research Time: 2019.10-2025.12

Schedule:

Phase I (up to primary endpoint)	FPI:	2019.12
	LPI:	2020.12
	LPLV	2021.03
	SAR	2021.07
	Publication	2021.07
Phase II (up to [illegible]	LPLV	2025.09
	CSR	2025.12

Endpoints: Primary endpoint: objective response rate (ORR)

Secondary endpoints: pathological down-staging rate, pathological complete response rate, R0 resection rate, EFS, DFS, PFS, OS, adverse events, health-related life quality

Exploratory endpoints: Potential biomarkers, tumor microenvironment dynamics.

Statistical method: All data will be presented on basis of the full analysis set. Continuous variables will be summarized according to the number of observed value, average value, standard deviation, median value, minimum value and maximum value; and categorical variables will be summarized by frequency counts and percentages for each category. The median time to events (death and progression) or censoring time, quartile Kaplan-Meier estimates and their 95% confidence intervals will be used to summarize EFS， OS, DFS and PFS.

Follow-up: After treatment, the patients will be followed up in the outpatient every 3 months until the disease recurs or 2 years later. After 2 years, the patients will be followed up in the outpatient every 6 months until the disease recurs or 5 years later. The outpatient follow-up examination includes symptom check, physical examination, chest CT scan, tumor biomarker examination, upper

	abdominal ultrasound examination, systemic ECT in case of bone pain, and cranial MRI in case of headache, vomiting, bilateral limb inequality, and walking instability, etc. In addition, the telephone follow-up will be made every 3 months from year 3 to 5 after treatment to obtain the data on disease recurrence and overall survival of patients.

CONTENTS

1. Background and Rationale 24
1.1 Epidemiology of Lung Cancer 24
1.2 Non-Small Cell Lung Cancer 24
1.3 Current Status on Treatment of Resectable NSCLC 24
1.4 Gene Mutation and Non-small Cell Lung Cancer 25
1.5 Therapeutic Target for Lung Adenocarcinoma: EGFR 25
1.6 EGFR Tyrosine Kinase Inhibitor (EGFR-TKI) 25
1.7 Afatinib 26
1.8 Current Status of Treatment with EGFR-TKI 26
1.9 Neoadjuvant TKI therapy 26
2. Research Objectives 27
3. Trial Content 27
3.1 Design Pattern 27
3.2 Selection of Patients 27
3.3 Sample Size 29
3.4 Study Schedule 29
3.5 Therapy Plan 30
3.6 Research Process 33
3.7 Indicators of Research 37
3.8 Termination of Study 40
4. Ethical and Legal Matters 40
4.1 Independent Ethics Committee (IEC) or Institutional Review Committee (IRB) 40
4.2 Ethical Guidance for the Study 40
4.3 Instructions to Participants and Informed Consent 41
4.4 Privacy Protection 41
4.5 Researcher Training 41
5. Research Supervision 41

6. Statistical Analysis......42
6.1 Sample Size......42
6.2 Description on Outcome Variables Related to Purpose and Assumptions......42
6.3 Description on Analysis Set......43
6.4 Statistical Analysis Methods......43
7. Adverse Events......45
7.1Considerations/Precautions......45
7.2Monitoring of Adverse Events......45
7.3 Definition of Adverse Event......45
7.4 Report of Serious Adverse Events/Pregnancy......48
7.5 Non-Serious Adverse Reactions to Drugs......49
8. Data Processing and Preservation......49
8.1 Case Report Form (CRF)......49
8.2 Preservation of Research Documents, Case Report Forms and Records......49
8.3 Archiving......50
8.4 Establishment of Database......50
9. References:......50
10. Appendixes......53
Appendix I ECOG Scoring Criteria......53
Appendix II TNM Classification – International Lung Cancer Staging Standard (8th Edition)......55
Appendix III NYHAFunctional Classification......58
Appendix IV Common Terminology Criteria for Adverse Events of NCI (CTCAE v4.0)......59
Appendix V EORTC QLQ-C30 (version 3) Life Quality Questionnaires......70

1. Background and Rationale

1.1 Epidemiology of Lung Cancer

Primary bronchial lung cancer is one of the most common malignant tumors. According to the latest data reported in 2018, lung cancer ranks first in terms of the incidence (11.6%) and the mortality (18.4%) among malignant tumors.[1] Similarly in our country, the data collected in 2015 show that lung cancer ranked first as well in terms of the incidence (19.6%) and the mortality (26.0%) among all malignant tumors, with an obvious increase compared with the incidence (13.0%) and the mortality (18.0%) in 2007. As analyzed by gender, lung cancer ranks first in terms of the incidence and the mortality among diseases suffered by male, while it ranks second in terms of the incidence (next only to breast cancer), and first in terms of the mortality among diseases suffered by female.[2-4] About 25% of the lung cancer patients all over the world live in the Asia-Pacific region, and lung cancer has become a powerful killer in the world, especially in Asia. According to the latest statistics of SEER (Surveillance, Epidemiology, and End Results) database, the 5-year overall survival rate of lung cancer is 19.4%.[5]

1.2 Non-Small Cell Lung Cancer

Non-small cell lung cancer (NSCLC) is the most common pathological type of all lung epithelial cancers except small cell lung cancer (SCLC), accounting for 80-85% of all lung malignant tumors.[6] The most common histological subtypes of NSCLC include adenocarcinoma and squamous cell carcinoma. However, any type of non-small cell lung cancer may occur under the effect from abnormal histological variation.[6] The TNM staging system proposed by the American Joint Committee on Cancer (AJCC) has been widely adopted by all countries in the world. The 8th edition of TNM staging system was promulgated on January 1, 2017, which was recognized as the best staging standard for prognosis of tumors.[7] Additionally, it was also recommended for the clinical research in the Guidelines (2017 edition) newly issued by the National Comprehensive Cancer Network (NCCN). According to the staging criteria of lung cancer by TNM (Version 8), the 5-year survival rate is 80% in NSCLC patients at pathological stage I, 60% in patients at stage II, and 41% in patients at stage IIIA.[8] Compared with small cell lung cancer, non-small cell lung cancer is less sensitive to radiotherapy and postoperative adjuvant chemotherapy, and the overall 5-year survival rate of which is less than 15%.[9]

1.3 Current Status on Treatment of Resectable NSCLC

The best treatment for lung cancer is still radical resection, which is indicated for patients at stage I-II and partial patients at stage III. However, the surgery is only applicable to 20-25% of NSCLC patients.[10] In most patients, the cancer may develop into the advanced stage or be found with distant metastasis when it is diagnosed confirmatively, and cannot be resected surgically. More than 60% of patients after surgical resection will develop recurrence, mainly due to micrometastasis. Adjuvant or neoadjuvant chemotherapy is the reasonable method to eradicate micrometastasis and minimize the risk of recurrence. A LACE meta-analysis of adjuvant therapy and neoadjuvant therapy trials, in which cisplatin-based chemotherapy has been analyzed, shows that the patients have a survival benefit of 5% within 5 years from the initiation of adjuvant or neoadjuvant chemotherapy, among which the patients at stage II and IIIA have the greatest survival advantage.[34] In the past 20 years, the systematic therapy has developed from a single empirical method with cytotoxic drugs to a targeting therapy or

immunotherapy with multiple options and better tolerance.[11, 12] However, the overall survival rate of NSCLC patients remains very low at present, and the continuous development and improvement of new drugs, technologies and treatment patterns are still needed to overcome the difficulty in treatment of lung cancer.

1.4 Gene Mutation and Non-small Cell Lung Cancer

Gene is the genetic information of organisms and determines the structure and functions of cells. Lung cancer is a heterogeneous disease, in which gene mutation plays a vital role with regard to its occurrence and development. The change of tumor genome is extremely complicated. Genetic variation information of genome mainly includes: single nucleotide variation at DNA level, chromosome copy number variation (including gene amplification and large-segment deletion), gene rearrangement,etc.[13-15] transcriptome variation at RNA level, and epigenetic change caused by gene mutation. Driver gene (proposed by Weinstein in 2002 [16]) refers to some molecular changes that play a key role in inducing and maintaining malignant tumors. In recent 10 years, a great progress has been made in studying lung cancer driver genes. In 2015, The Cancer Genome Atlas (TCGA) had fully reported the results of genome landscape on lung adenocarcinoma and squamous cell carcinoma. [13-15] About 60% of the driver genes in lung adenocarcinoma had been identified, including more than 10 genes such as KRAS, EGFR and BRAF. In lung squamous cell carcinoma, the driver genes such as TP53, FGFR and SOX were found. Gene fusion and gene rearrangement had been achieved in genes such as KRAS, EGFR and FGFR, which became the potential therapeutic targets, and brought opportunities for targeted therapeutic interventions. [17-20]

1.5 Therapeutic Target for Lung Adenocarcinoma: EGFR

Epidermal growth factor receptor (EGFR) belongs to receptor tyrosine kinases (RTKs) of HER/erbB family, including HER1 (EGFR/erbB1), HER2 (neu, erbB2), HER3 (erbB3) and HER4 (erbB4). EGFR gene is located on the short arm of chromosome 7 (7p11.2) and encodes type I transmembrane growth factor receptor with tyrosine kinase activity. [21] After binding to its ligand, it can induce autophosphorylation of the receptor cytoplasmic domain, [22] and can activate downstream EGFR signal pathways such as RAS-RAF-MEK-MAPK pathway, PI3K-PTEN-AKT pathway, signal transducer and transcriptional activator (STAT), [23] thus promoting effects such as cell proliferation, angiogenesis, metastasis and anti-apoptosis finally. [24] Tyrosine kinase activity of EGFR is regulated by carcinogenic mechanisms such as EGFR mutation, copy number increasing, and EGFR protein overexpression. [25] Receptors and ligands of EGFR family also mediate complex interactions between tumor cells and tumor microenvironment. Inappropriate activation of EGFR tyrosine kinase can inhibit apoptosis of tumor cells and promote tumor progression. [26] EGFR may also interact with integrin pathway, [27,28] activate matrix metalloproteinase, alter cell adhesion, stimulate cell migration and invasion, and promote metastasis. [29] EGFR mutations occur mainly in the coding region of tyrosine kinase, most of which are concentrated in exons 18-21. The most common mutations in lung adenocarcinoma are deletion mutation at exon 19 (about 44%) and L858R mutation at exon 21 (about 41%). [30] Mutation of EGFR tyrosine kinase domain could lead to conformational instability, constitutive activation of kinase activity and activation of downstream signal pathways, [31,32] thus causing the occurrence and development of lung cancer.

1.6 EGFR Tyrosine Kinase Inhibitor (EGFR-TKI)

EGFR tyrosine kinase inhibitor (TKI) can competitively inhibit the combination of ATP and EGFR tyrosine kinase domain, thus to prevent growth, proliferation, invasion, angiogenesis and metastasis of EGFR activated mutant tumor cells by blocking downstream signal pathways. At present, TKIs include generation-I drugs (gefitinib, icotinib, erlotinib), generation-II drugs (afatinib, dacomitinib), generation-III drugs (osimertinib, avitinib) and generation-IV drugs (e.g. EAI045) in preclinical studies.

1.7 Afatinib

Afatinib (trade name: Giotrif) is a second-generation TKI, researched and developed by Boehringer Ingelheim of Germany, which can act as the irreversible covalent inhibitors of ErbB family, characterized with lasting effect and significantly longer progression-free survival (PFS) compared with the generation-I TKI-based drugs (reversible inhibitors). [33] Afatinib has been approved to treat non-small cell lung cancer with positive EGFR gene mutation in more than 70 countries worldwide.

1.8 Current Status of Treatment with EGFR-TKI

According to the latest guidelines of NCCN, TKIs (erlotinib, gefitinib, afatinib and osimertinib) is the first-line therapy for advanced NSCLC with positive EGFR mutation. It has been found in studies that TKIs therapy can also benefit patients with resectable NSCLC. In a randomized, open-Label, phase III clinical trial (ADJUVANT/CTONG1104), total 222 EGFR mutation-positive patients with NSCLC at stage II-IIIA were enrolled [35] and divided into the experiment group (administrated with gefitinib at a dosage of 250 mg per day for 2 years) and the control group (administrated with vinorelbine at a dosage of 25 mg/m^2+ cisplatin at a dosage of 75 mg/m^2) at a rate of 1:1, to receive 4 cycles of treatment, with 3 weeks as one cycle. The result showed that in the experiment group, the median disease-free survival (DFS) was significantly higher (36.5 vs. 28.7, $P<0.01$), and the incidence of toxic and side effects was lower, compared with those in the control group. Another randomized phase II clinical trial showed that IIIA-N2 patients who received postoperative chemotherapy combined with adjuvant gefitinib had got better DFS (39.8 vs. 27.0, P=0.014) and 2-year DFS (78.9% vs. 54.2%) than those who received chemotherapy alone. [36] In addition, there are a number of trials that are ongoing to investigate the TKIs adjuvant therapy for resectable NSCLC (NCT00373425, NCT03983811, NCT01843647, and NCT03656393, etc.).

1.9 Neoadjuvant TKI therapy

Neoadjuvant therapy could bring treatment effects such as tumor shrinking, improving the complete resection rate, eliminating micrometastases and reducing the risk of recurrence. It has been reported that the neoadjuvant TKI therapy could achieve the downstaging of tumor or even the complete response in the locally advanced (IIIA-N2) NSCLC patients. [37,38] The results of a neoadjuvant TKI clinical trial, which is the first randomized, controlled study to confirm the application of EGFR-TKI in neoadjuvant and adjuvant phases, were reported in European Society of Medical Oncology (ESMO) Conference. In this EMERGING trial, 72 EGFR mutation-positive patients with NSCLC at stage IIIA-N2 were included and divided into the erlotinib group (administrated with erlotinib at a dosage of 150mg per day for 6 weeks before operation and 1 year after operation) and the chemotherapy group (administrated with gemcitabine at a dosage of 1250mg/m^2+ cisplatin at a dosage of 75mg/m^2 for two cycles before operation and two cycles after operation, with every 3 weeks taken as 1 cycle). The current results show that in the erlotinib group, the median PFS has been significantly prolonged compared with that in the chemotherapy group (21.5 vs 11.4 months, $P<0.001$), but the objective response rate (ORR) does not improve significantly (54.1% vs 34.3%, P=0.092). [39]

Afatinib, as a generation-II TKIs, theoretically has better neoadjuvant effect than the generation-I TKIs. At present, there is only one single-arm phase II clinical trial (ASCENT) on neoadjuvant afatinib in treatment of stage III EGFR mutation-positive NSCLC. The study found that the ORR of neoadjuvant afatinib was as high as 75%, and the median PFS was 34.8 months, but the sample size was small (only 13 cases). [40] However, no more studies are available so far to confirm the efficacy and safety of neoadjuvant afatinib in the treatment of resectable stage III EGFR mutation-positive NSCLC, and there is a lack of studies based on the Chinese population. Given that the neoadjuvant therapy has delayed the operation time and there is a potential risk of cancer progression, more data are needed to perform evaluation.

2. Research Objectives

To evaluate the efficacy and safety of neoadjuvant Afatinib in the treatment of stage III EGFR mutation-positive lung adenocarcinoma.

3. Trial Content

3.1 Design Pattern

A single-arm, open-Label, single-center, phase II clinical trial

3.2 Selection of Patients

EGFR mutation-positive lung adenocarcinoma patients at stage III (version 8)

3.2.1 Inclusion Criteria

- Lung adenocarcinoma patient with EGFR sensitive mutation as confirmed by needle biopsy;
- At stage III (TNM Staging, Version 8) as identified by chest CT, PET-CT or/and EBUS;
- No systemic metastasis (confirmed by head MRI, whole body bone scan, PET-CT, liver and adrenal CT, etc.);
- With the feasiblility to receive radical surgery (radical lung lobectomy+systematic lymph node dissection);
- Good lung function that could tolerate surgical treatment;
- Aged 18- years;
- At least one measurable tumor foci (the longest diameter measured by CT shall be > 10 mm);
- Other major organs shall function well (liver, kidney, blood system, etc.):
  - Hemoglobin ≥9.0 g/dL (which can be maintained or exceeded by means of blood transfusion);
  - Red blood cell count ≥2.0×10^9/L;
  - Absolute neutrophil count (ANC) ≥ 1.0× 10^9/L;
  - Platelet count ≥ 100× 10^9/L;
  - Total bilirubin is within the normal limit;
  - Alanine transaminase, glutamic oxaloacetic transaminase and alkaline phosphatase ≤2.5 times the upper limit of normal value;

- Creatinine ≤ 2.0 mg/dL; and creatinine clearance rate ≥ 60 ml/min;
- The international normalized ratio (INR) of prothrombin time and the activated partial thromboplastin time (APTT) of patient who has not received anticoagulant therapy shall be ≤ 1.5 times the upper limit of normal value. The patient who has received full or parenteral anticoagulant therapy shall be allowed to participate in the clinical trial only when the dosage of anticoagulant drugs is stable for at least 2 weeks, and the results of coagulation test are within the limits for local treatment.

- ECOG PS score shall be 0-1;
- The child-bearing female must undergo pregnancy test within 7 days before starting the treatment and the result shall be negative. Reliable contraceptive measures, such as intrauterine device, contraceptive pill and condom, shall be adopted during the trial and within 30 days after completion of the trial. The child-bearing male shall use condom for contraception during the trial and within 30 days after completion of the trial;
- The patient shall sign the Informed Consent Form.

3.2.2 Exclusion Criteria

- The patient has undergone any systemic anti-cancer treatment for NSCLC, including surgical treatment, local radiotherapy, cytotoxic drug treatment, targeted drug treatment and experimental treatment, etc.;
- The patient suffered from other cancers besides NSCLC (except cervical carcinoma in situ, cured basal cell carcinoma and bladder epithelial tumor [including Ta and Tis]) within 5 years before the trial;
- The patient suffers from any unstable systemic disease (including active infection, uncontrolled hypertension, unstable angina pectoris, angina pectoris that starts to attack within the last 3 months, congestive heart failure [≥ Grade II specified by New York Heart Association (NYHA)], cardiac infarction (6 months before enrollment), severe arrhythmia and liver, kidney or metabolic diseases that requires drug treatment;
- The patient is a carrier of active hepatitis B, hepatitis C or HIV;
- The patient suffers from severe or new-onset gastrointestinal diseases with diarrhea as the main symptom;
- The patient is receiving the P glycoprotein inhibitor therapy;
- The patient has had or is currently suffering from cardiovascular malformation;
- The patient has had or is currently suffering from interstitial lung disease;
- The patient had undergone other major systemic operations or suffered from severe trauma within 3 months before the trial;
- The patient is allergic to afatinib or its any excipients;
- The patient suffers from nervous system diseases or mental diseases and cannot keep compliance with the trial;
- The patient has any malabsorption condition;

- The female patient is in pregnancy or lactation period;
- There are any conditions under which the investigator considers the patient is not suitable to be enrolled.

3.2.3 Withdrawal from Trial

A withdrawal case refers to the patient who have terminated the neoadjuvant therapy due to various reasons during the clinical research. The patient will be withdrawn from the trial under the following conditions:

- The withdrawal is required by the patient himself or his legal representative;
- Continued participation of the patient in the study, as considered by the investigator, will be harmful to his/her health;

The patient must be withdrawn from the trial under any one of the following conditions:

- The patient has formed severe allergic reaction to the chemotherapy drugs, such as exfoliative rash or hypersensitivity of grade 3 to 4;
- There is any other serious adverse reaction that the principal investigator (PI) or the investigator designated by PI believes it will need to suspend the treatment;
- The patient has shown very poor compliance;
- The results of β-HCG test to the patient suggest a pregnancy. The pregnancy event shall be reported in the clinical trial pregnancy report form;
- During the study, the patient has developed other diseases concurrently. It is believed by the investigator that the disease will obviously affect the evaluation of clinical situation to the patient, and it is necessary to terminate the treatment with this regimen;
- The patient has been found with any other malignant tumor that needs treatment;
- The patient gets lost to the follow-up visit;
- The patient has taken prohibited drugs or other substances that may lead to toxic reactions or lead to deviation of research results according to the judgment of the investigator;
- Death of the patient.

The reason for withdrawal shall be recorded in the case report form and the patient's medical record for any patient who has withdrawn from the trial.

It needs to follow up all patients who have withdrawn due to adverse events or abnormal laboratory test results until the adverse events are solved or become stable, and to record the subsequent results of such events. If a patient dies during the trial treatment or within 28 days after the completion of the experiment treatment, the investigator shall notify it to the Sponsor. The cause of death must be recorded in detail in the serious adverse events (SAE) report form and present the report form to the ethics department within 24 hours.

3.3 Sample Size

A total of 47 patients will be included in this trial.

3.4 Study Schedule

Assuming 4 patients are enrolled each month, it will take about 12 months for all patients to be enrolled. The first patient will be enrolled in Jan of 2020, and the enrollment of all patients is expected to be completed in December of 2020. The first stage will be from the enrollment of patients up to the completion of ORR evaluation to all patients. The primary endpoint, ORR, will be evaluated 3 months after the last patient is enrolled into the group, i.e. in March of 2021 (15th month later), and the preliminary results will be published in July of 2021 (within 4 months after ORR evaluation results are released).

The second stage is from the surgery treatment up to the completion of follow-up procedure. The evaluation of pathological down-staging rate, pCR and R0 resection rate can be completed one month after the operation of the last patient, i.e. in April of 2021. EFS, PFS and DFS will be basically determined (with reference to EMERGING results) at the 48th month (i.e. December of 2023, 36 months after the last patient has been enrolled in the group). OS follow-up time will be about 60 months. The last patient will complete the survival visit in September of 2025, and all data statistics, analysis and publication work with regard to secondary endpoints will be completed in December of 2025. Therefore, the total research period will be about 72 months.

3.5 Therapy Plan

3.5.1 Therapy Regimen

The neoadjuvant therapy stage: the enrolled patients will take afatinib at a dosage of 40mg per day for 8-16 weeks; and chest CT evaluation will be performed within 3 weeks after completion of the therapy.

Surgical treatment stage: the patients who have a response to afatinib therapy (CR+PR) and the patients who have no response to afatinib therapy but can still receive surgical treatment (SD and PD) will receive radical lobectomy+systematic lymph node dissection on the 22th post-therapy day.

Adjuvant treatment stage: the patients with CR, PR or SD who have undergone the operation will take afatinib at a dosage of 40mg per day for 1 year.

The patients with SD or PD who cannot receive operation and the patients with PD who have undergone the operation will be transferred into medical oncology or/and radiation oncology and receive comprehensive therapy (chemotherapy or/and radiotherapy, the regimen is designed by oncologist and radiologist.

3.5.2 Treatment Flow Chart

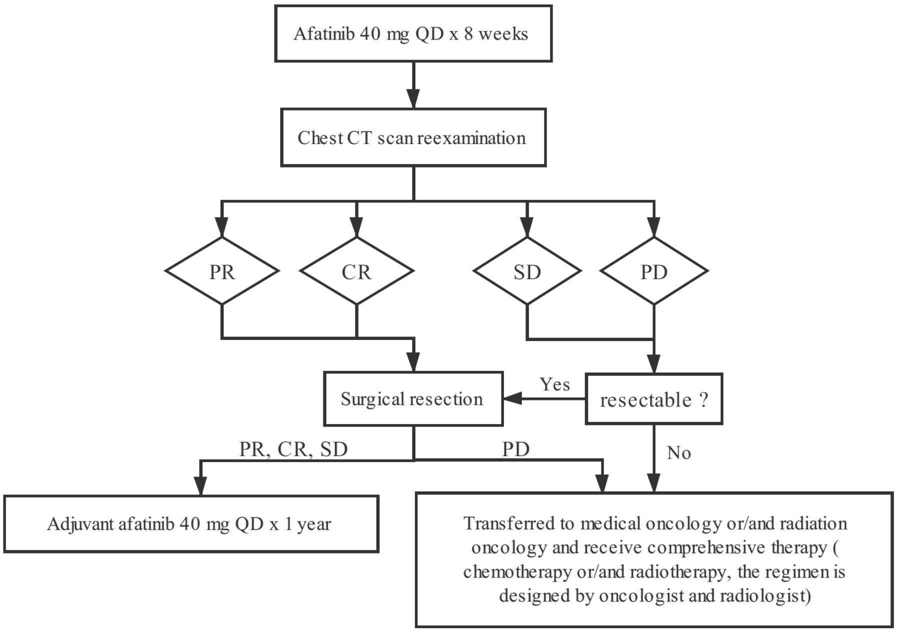

3.5.3 Dosage Adjustment and Disposal of Missed Dose

Dosage adjustment due to adverse reactions

The symptomatic drug-related adverse reactions (such as those accompanied by severe/persistent diarrhea or skin-related adverse reactions) can be controlled by suspending the treatment and reducing the dosage of the test drug as listed in Table 1. (Please see the section "Adverse Reactions" in the Investigator Brochure or Instruction; and for more details on disposal of specific drug-related adverse event (AE), please refer to the section "Precautions" in the Investigator Brochure or Instruction.)

Table 1 – Dosage Adjustment for Adverse Reactions

CTCAE[a] Drug-related Adverse Events	Recommended Dosage of Afatinib	
Grade 1 or 2	No suspension [b]	No dosage adjustment
Grade 2 (prolonged [c] or intolerant) or ≥ Grade 3	To suspend medication up to restoration to grade 0/1 [b]	To reduce the dosage progressively by 10 mg [d]

[a] Common Terminology Criteria Adverse Events (Version 4.0) of The American National Cancer Institute (NCI).

[b] When diarrhea occurs, the anti-diarrhea drugs (such as loperamide) shall be used immediately, and in the event of persistent diarrhea, the medication shall be kept up to the cessation of diarrhea.

[c] Diarrhea has lasted for more than 48 hours and/or rash has lasted for more than 7 days.

[d] If the patient cannot tolerate the dosage of 20mg per day, permanent termination of the medication shall be considered.

Missed Dose

If the dose is missed once, the patient shall take the test drug as soon as possible when he/she remembers on the same day. However, if less than 8 hours have been left before the next administration, there will be no need to supplement the missed dose.

3.5.4 Concomitant Therapy and Smoking

All concomitant medications and treatments (including start/end dates and indications) must be recorded in the patient's original data and the Case Report Form (CRF). It is recommended that all patients give up smoking during treatment.

3.5.5 Concomitant Medication

3.5.5.1 Drugs Not Allowed to be Used

- Bevacizumab, other TKIs and any drugs targeting VEGF, VEGFR, etc. (including those registered or under study).
- Research drugs (e.g. antibiotics, antiemetics and other drugs under research).
- Any Chinese herbal medicine treatment indicated with anti-tumor efficacy.

3.5.5.2 Drugs Allowed to be Used

- Unconventional therapy (e.g. non-anticancer Chinese herbal medicine therapy or acupuncture) and vitamin/trace elements are also allowed to be used provided that they cause no effect on the observation of the research endpoints, depending on decision of the investigator.
- The patients are allowed to receive palliative treatment and supportive treatment for the original diseases.
- Folic acid, vitamin B12 and corticosteroids are allowed to be used preventively to reduce toxic reactions.

3.5.6 Treatment Compliance

A record shall be made in the Case Report Form with regard to the dosage and administration time of Afatinib, the drug name, dosage and administration time of the drug used in platinum-doublet chemotherapy for each participant in each course of treatment, as well as the reasons for delayed administration, dosage reduction or missed dose.

The compliance of a participant with the treatment and protocol includes his/her voluntary compliance with all aspects of the protocol. If the participant fails to make visits or take drugs on time, he or she could be withdrawn from the study according participant to the opinion of the principal investigator.

3.6 Research Process

3.6.1 Screening Period and Baseline Period

Screening period: within 7 days before commencement of neoadjuvant therapy

- To record/confirm the pathological type, mutation type and clinical tumor stage of the patient;
- To get the informed consent form signed.

Baseline period: within 7 days before the experiment group starts the neoadjuvant therapy

- To record demographic information, complete medical history and smoking history, including previous anti-cancer treatment.
- To perform complete physical examination (PE), including ECOG performance status score, height, weight and detailed systematic examination.
- To check vital signs (including heart rate, blood pressure, respiratory rate and body temperature).

- To record all concomitant diseases, concomitant drugs and their indications.
- Blood routine examination: hemoglobin, hematocrit, platelet count, white blood cell count and classification, including absolute counts of neutrophils and lymphocytes.
- Whole blood biochemical examination: including blood sugar, calcium, phosphorus, sodium, potassium, chlorine, creatinine, blood urea nitrogen (BUN), total protein, albumin, alanine transaminase (ALT), aspartate transaminase (AST), alkaline phosphatase, total bilirubin, etc.
- Tumor biomarkers.
- To perform serum pregnancy tests for all female patients of child-bearing age, including those whose partners have undergone sterilization operation. Pregnancy tests are not required for postmenopausal female patients who have been menopausal for at least one year and for the female patients who have undergone sterilization operation.
- Routine urine examination: including urine specific gravity, pH value, urine sugar, urine protein and occult blood.

3.6.2 Neoadjuvant Treatment Period

The enrolled patients will take afatinib at a dosage of 40mg per day for 8-16 weeks. Adverse events and concomitant medication of patients will be recorded during the period. The blood routine examination results, blood biochemical examination results and tumor biomarkers of the patients will be evaluated at the end of Week 4 and the last neoadjuvant cycle (4 weeks per cycle).

3.6.3 Imaging Evaluation after Neoadjuvant Therapy

Chest CT examination will be performed within 3 weeks after completion of treatment to assess the response of patient (according to RECIST1.1) and to determine whether surgical resection is feasible.

3.6.4 Surgical Treatment Period

The patients who have a response to afatinib therapy (CR+PR) and the patients who have no response but could still receive the surgical treatment (SD and PD) will receive radical lobectomy and systematic lymph node dissection after chest CT re-examination.

3.6.5 Adjuvant Treatment Period

The patients with CR, PR and SD who have undergone the operation will take afatinib at a dosage of 40mg per day for 1 year;

The patients with SD or PD who cannot receive operation and the patients with PD who have undergone the operation will be transferred into medical oncology or/and radiation oncology and receive comprehensive therapy (chemotherapy or/and radiotherapy, the regimen is designed by oncologist and radiologist.

3.6.6 Follow-up after Treatment

Upon completion of treatment, the patients will be followed up every 3 months until the disease recurs or 2 years later. After 2 years, the patients will be followed up every 6 months until the disease recurs or 5 years later. The outpatient follow-up examination includes symptom check, physical examination, chest CT scan, tumor marker examination, upper abdominal ultrasound examination, systemic ECT in case of bone pain, and cranial MRI in case of headache, vomiting, bilateral limb inequality, and walking instability, etc. In addition, the telephone follow-up will be made every 3 months from 3 to 5 years after treatment to obtain the data on disease recurrence and overall survival of patients.

3.6.7 Unscheduled Visits

Unscheduled visits shall be made according to the clinical needs, and relevant information shall be recorded in CRF and original data, including laboratory examination abnormalities and adverse events of clinical significance. If multiple laboratory tests have been carried out within the same day, only the latest results need to be recorded in CRF. However, all abnormal values found in the repeated laboratory tests shall be recorded in CRF.

3.6.8 Follow-up of Patients with Relapse or No Relapse after Study (5 Years after Operation)

The patients will be contacted once every 3 months (by on-site visit or phone call) to obtain the information about the overall survival and postoperative treatment of the patients. The follow-up visit shall be made every 3 months until the patient dies. The following information should be obtained during each follow-up visit:

- Survival status.
- If dead, the date and cause of death shall be recorded in detail.
- Disease status and relapse, in the event of relapse, the relapse date shall be recorded in detail (for those patients who have not got relapse in the last follow-up).
- The subsequent postoperative anti-cancer treatment (including reoperation, chemoradiotherapy and immunotherapy after relapse) shall be recorded in detail.

3.6.9 Flow Sheet of Research

	Screening period	Baseline period	Neoadjuvant treatment period			Evaluation	Operation	Adjuvant treatment period		Follow-up
Number of weeks	-1~0	-1~0	1-16	-8	2-16	9-10 or 17-18	11 or 19		Monthly assessment[1]	
Informed Consent Form	X									
Inclusion/Exclusion Criteria		X								
Medical history (including smoking)	X									
Physical examination		X				X				
Vital signs		X				X				
ECOG score		X								
ECG		X								
Chest CT						X				
Cranial CT or MRI		X								
HIV test		X								
Blood routine		X							X	
Blood biochemistry		X							X	
Urine routine		X								
Tumor biomarker		X				X				
Pregnancy test (if applicable)		X								
Concomitant disease		X								

Concomitant medication										
Afatinib			X							
Comprehensive therapy								2		
Tumor assessment (RECIST)		X				X				
Surgery							X			
Safety Assessment/Record [3]			X			X	X		X[3]	
EORTC-QLQ-C30&LC13									X	
Postoperative follow-up and survival follow-up										X

1. The patients who receive chemotherapy will be evaluated at the end of each cycle, while the patients who take afatinib will be evaluated according to the clinical symptoms.

2. The patients with SD or PD who can not receive operation and the patients with PD who have undergone the operation will be transferred into medical oncology or/and radiation oncology and receive comprehensive therapy (chemotherapy or/and radiotherapy, the regimen is designed by oncologist and radiologist.

3. All adverse events shall be collected from the signature of ICF by participants up to 30 days after the last administration of afatinib or platinum-doublet chemotherapy. Then only SAE related to afatinib or platinum-doublet chemotherapy will need to be collected up to the completion of study.

3.7 Indicators of Research

3.7.1 Primary Endpoint

Objective response rate (ORR)

According to RECIST1.1, ORR refers to the proportion of patients who have achieved a tumor shrinkage to certain an extent and had it maintained for a certain period of time, including patients with complete response (CR) and partial response (PR). Complete response: all target lesions disappear, and the short diameter of any pathological lymph node (including target nodules and non-target nodules) must be reduced to a length of <10 mm. Partial response: the

sum of target lesion diameters is reduced by at least 30% from baseline level. In this study, ORR is defined as the proportion of patients who have completed the 8-to-16-week treatment with afatinib before operation and have achieved CR or PR as confirmed by CT evaluation after 3 weeks in all patients.

3.7.2 Secondary Endpoints

Pathological downstaging rate: It is defined as the proportion of patients who have completed the 8-week treatment with afatinib before operation and have achieved a T stage downing of the tumor as confirmed by CT evaluation after 3 weeks in all patients who have completed the treatment.

Pathological complete remission (pCR): It is defined as the proportion of patients with no residual carcinoma in primary tumor lesions and lymph nodes under microscope with HE staining in all patients who have completed the treatment.

R0 resection: It is defined as the proportion of patients with negative surgical margin and no residual found under microscope after resection in all patients who have completed the treatment.

Event-free survival (EFS): It is was defined as the time from the first administration of afatinib in this study to any progression of disease precluding surgery, progression or recurrence of disease after surgery, progression of disease in the absence of surgery, or death from any cause (including any cause of death in the event of no progression) as recorded in CRF.

Progression-free survival (PFS): It is defined as the time from the first administration of afatinib in this study to the disease progression or death.

Disease free survival (DFS): It refers to the time from radical surgery to relapse or death of a participant due to disease progression.

Overall survival (OS): It is defined as the time from random enrollment to death of participant due to any cause.

Treatment-related adverse events: It refers to the number of adverse events related to afatinib monotherapy or platinum-based chemotherapy as evaluated according to CTCAE v4.0.

Health related quality of life (HRQol): The assessment is made according to the Quality of Life Scale for Lung Cancer Patients (EORTC-QLQ-C30 & LC13, Version 3).

3.7.3 Exploratory Endpoints

Participants enrolled in study will undergo the same laboratory correlate studies on tumor biopsy, resection specimen and serum samples to explore potential biomarkers associated with therapy response and tumor microenvironment dynamics during therapy.

3.7.3.1 Tumor tissue samples

Fresh specimens from lung tumor, lymph node, tumor adjacent normal tissue before treatment (in screening period, by biopsy), on treatment (biopsy) and post-treatment surgery shell be collected. Where possible, and after a consent form has been signed, attempts will be made to coordinate diagnostic and study biopsies in screening period.

3.7.3.2 Peripheral blood samples

Blood samples (10 mL) will be drawn at the time points identified on the study calendar. Time points include: screening period, before drug administration in each cycle and pre-surgery evaluation. Whole blood will be collected in serum separator tubes and stored at -70°C or below until transfer for analysis. Viable PBMCs will be stored in cryopreservation medium, at 5e6-1e7 per vial, in liquid nitrogen.

3.7.3.3 Methods of analysis

(1) Immunohistochemistry/multiplex immunofluorescence

Tumor and lymph node biopsies will be stained using commercially available and locally developed monoclonal antibodies. Analyses may include phenotypes of infiltrating immune cell populations including but not limited to CD3, CD4, FOXP3, CD25, CD8, CD68, CD56, CD20, CD45RO and granzyme B. Peritumoral versus intra-tumoral infiltrates will be scored, since these staining patterns have been shown to correlate with clinical outcomes. Immunohistochemical analysis of exploratory markers will focus on areas where the pathology co-investigators have established expertise, including but not limited to: the B7 family ligands PD-L1 (B7-H1), PD-L2 (B7-DC), B7-H3 and B7-H4, as well as inhibitory receptors on lymphocytes, including PD-1, 2B4, LAG-3, BTLA, and Tim-3; these cell surface molecules are candidates for therapeutic combinatorial antibody blockade. Expression of the ligands for Tim-3, BTLA and 2B4 (galectin 9, HVEM, and CD48, respectively) may also be evaluated as well as cytokine expression.

(2) Single-cell RNA-seq and TCR/BCR-seq

Tissue samples and blood samples will be isolated and transported rapidly to the research facility. Tissues were transported in a sterile culture dish on ice (4 °C) to remove the residual tissue storage solution, then minced into 1-3 mm3 pieces in another culture dish. All subsequent steps were performed following the standard manufacturer protocols. After reverse transcription and cell barcoding, emulsions were broken and cDNA was isolated and purified, followed by PCR amplification. Amplified cDNA was then used for both 50 gene expression library construction and TCR V(D)J and BCT V(D)JC targeted enrichment performed with the Chromium Single Cell V(D)J(C) Enrichment Kit, followed by V(D)J library construction. For RNA-seq library construction, amplified cDNA was fragmented and end-repaired, double-sided size-selected, PCR-amplified with sample indexing primers, and double-sided size-selected. For TCR-seq/BCR-seq library construction, TCR/BCR transcripts were enriched from amplified cDNA by PCR. Subsequently, enriched PCR product was fragmented and end-repaired, size-selected, PCR-amplified with sample-indexing primers, and size-selected. Libraries prepared according to the manufacturer's user guide were then purified and profiled for quality assessment. Single-cell RNA and TCR/BCR V(D)J(C) libraries were sequenced by an Illumina HiSeq X-Ten sequencer with 150 bp paired-end reads

(3) Spatial transcriptomics

The surgically resected tumor tissue was immediately submerged in MACS Tissue Storage Solution and sent to the laboratory for processing as soon as possible. Then, the tissue is gently washed with cold phosphate-buffered saline (PBS) and cut into about 6.5-mm3 pieces (bulks)

according to the experimental design. All subsequent steps were performed following the standard manufacturer protocols.

(4) Bulk RNA-seq

RNA was isolated from fresh tissues, and then extracted using the RNeasy PowerLyzer Tissue & Cells Kit (QIAGEN) and quality was assessed with a 2100 Bioanalyzer System (Agilent Technologies). All samples were sufficiently high quality for TruSeq RNA Exome analysis (DV200 > 30%) and were prepared using the TruSeq RNA Exome Kit (Illumina) according to the manufacturer's instructions. After hybrid capture, cDNA libraries were pooled and sequenced on a HiSeq 2500 instrument (Illumina) using 2 × 150 bp paired-end reads with a target of 20–25 million reads per sample.

(5) Whole-exome sequencing

WES was performed per standard protocols using the Next-Generation Sequencing platform of TWIST bioscience company. Briefly, for DNA extraction according to the manufacturer's instructions and quantified. Libraries were generated. Subsequently, hybridization and capture were performed. After capture, libraries were amplified by PCR. Then, purified libraries were validated and quantified. Last, the enriched libraries were sequenced on the Nova6000 instrument of Illumina platform (Illumina).

(6) Flow cytometric analysis of tumor and lymph nodes

Cryopreserved viable single cell suspensions will be thawed, and cells will be stained with specific monoclonal antibodies to assess coordinate expression of co-regulatory molecules by tumor infiltrating lymphocytes, draining lymph node cells and tumor cells. Multicolor flow cytometric analyses will be conducted.

3.8 Termination of Study

This study may be terminated or suspended ahead of schedule if there is a sufficient reason, which includes but are not limited to the cases when:

- It is infeasible to evaluate the treatment plan since a major error has been found in the protocol of clinical trial.
- The Sponsor who has initiated the project requires to terminate the study (e.g. due to management reasons, and serious safety issues, etc.).

4. Ethical and Legal Matters

4.1 Independent Ethics Committee (IEC) or Institutional Review Committee (IRB)

According to GCP, Chinese laws and regulations and requirements of relevant organizations, all participating research centers shall obtain the approval from the corresponding ethics committee/institutional review committee prior to commencement of the study. When necessary, the approval from the ethics committee shall be extended, amended or reviewed, and transferred to the investigator.

4.2 Ethical Guidance for the Study

This study complies with the ethical principles set forth in the Declaration of Helsinki, the International Conference on Harmonization of Technical Requirements for Registration of

Pharmaceuticals for Human Use (IHC)/Quality Management Standards for Drug Clinical Trials, other relevant regulatory requirements, and Boehringer Ingelheim's policies on bioethics and human samples.

The procedures for operation, evaluation and documentation included in this research protocol are to ensure that the investigators will follow the guidelines of GCP and the guiding principles specified in the Declaration of Helsinki. The implementation of this research protocol will also follow the applicable laws and regulations of our country.

The investigators shall not modify the research protocol with no prior consent. However, in the event of emergency, the investigator may deviate from or change the research protocol in order to remove the risk factors for the participants, without the prior consent/support from the ethics committee/institutional review committee/Sponsor. The report on deviations or changes made thereby and their causes shall be submitted to the ethics committee/institutional review committee/Sponsor as soon as possible, and the proposals for modification of the protocol shall also be submitted if appropriate. The investigator must completely describe and explain all deviations or changes made to the research protocol.

4.3 Instructions to Participants and Informed Consent

The participants should be provided with the main information on the study and the Informed Consent Form (ICF). The investigator must provide the participants with written approval/agreement on the ICF from the ethics committee/institutional review committee as well as all other written information before the study starts. The approval documents from the ethics committee/institutional review committee, the approved instructions to participants and the Informed Consent Form must be filed together into the research document.

The ICF must be obtained before implementing any specific research steps. The date when a patient participates in the study and signs the ICF shall be recorded in the corresponding document of the participant.

4.4 Privacy Protection

All records that may identify the patients shall be kept confidential and will not be made public to the extent permitted by relevant laws and/or regulations.

Only the number and initials of a participant'name rather than his/her real name will be recorded in the Case Report Form (CRF). If the participant's name appears in any other document (e.g. pathological report), it must be covered in the copy of the document. The research report stored in the computer must comply with local laws on data protection. When the results of the study are published, the participant's identity will still be kept confidential.

The investigator will keep a list of names to identify participants' records.

4.5 Researcher Training

Before the first patient is included into the study, the representatives of Boehringer Ingelheim (BI) will review and discuss the research protocol and relevant documentation requirements with the working staff of research center, and provide training to them for any specific process of the protocol. The research institution shall ensure that all research staff involved in the study will receive appropriate training.

5. Research Supervision

If the protection to the privacy of patients meets the local requirements, the responsible supervisor (or designated personnel) will contact and visit the research institution regularly, and

shall be allowed to check various test records (including Case Report Form and other relevant data) as required.

During the study, the supervisor (BI representative) shall contact the research center regularly according to local regulations, including the requirements on the follow-up. If the investigators or other personnel of the center need information and suggestions on the implementation of the study, the representative of BI shall be available. The research center shall maintain the original data according to GCP or local regulations. The investigators in each center must abide by all terms and obligations in the research agreement or related documents of this research, and they must also comply with the filing requirements specified in the research agreement. The study is expected to start in December of 2019 and the last patient is expected to be enrolled in April of 2022.

6. Statistical Analysis

6.1 Sample Size

The primary endpoint of this research is ORR, which is taken to calculate the sample size.

Previous studies reported that the ORR of neoadjuvant erlotinib for NSCLC(EMERGING) at stage III-N_2 was 54.1%, the ORR in control group for neoadjuvant chemotherapy was 34.3%, [39] and the ORR of neoadjuvant afatinib for NSCLC (ASCENT) at stage III was 75%. [40] In view of that the small sample size of ASCENT trial (n=22) may lead to high ORR value, and afatinib has better therapeutic effect as the generation-II TKI than the generation-I TKIs, the ORR is estimated to be 60% in this study on basis of the treatment experience of our hospital.

The ORR value is expected to be 60% in this study, and the neoadjuvant chemotherapy group of EMERGING trial is taken as the historical control group. It is calculated with α=0.05 (two-tailed) and the power of test (1-β) =90%. According to the sample calculation of one-sample rate test, [41] when P_0 =34%, and P_1=60%, the sample size will be 42 as calculated, therefore, if the drop-off rate is 10%, the total sample size will be 47.

Based on this calculation method, after the first 20 patients (50% of the total patients) have received treatment and the response has been observed, we can perform failure analysis, which requires at least 7 patients with CR/PR to continue the trial. The basic principle is that under the substitution hypothesis (P_0=60%), the probability of observing a response in no more than 6/20 patients is <0.01 (0.0065). Therefore, if we take into account of Bayesian framework and assume a non-informative prior beta (1,1), the probability of ORR>60% will be < 0.01 (0.0065) in 6/20 responders.

If the trial proceeds until all 42 patients have been treated and evaluated, we will also be able to reasonably and accurately estimate the response rate (as shown in the following table, by using the Clopper-Pearson formula, and 95% exact confidence interval):

	# responders	Observed ORR (%)	95% CI (%)	Width of 95% CI
N=42	14	33.3	(19.6, 49.5)	30
	15	35.7	(21.5, 52.0)	30.5
	25	59.5	(43.3, 74.4)	31.1
N=47	16	34.0	(20.9, 49.3)	28.4
	28	59.6	(44.3, 73.6)	29.3

6.2 Description on Outcome Variables Related to Purpose and Assumptions

The following information in CRF will be evaluated through analysis:

EGFR mutation status, history of related diseases, body weight, disease characteristics, cancer treatment history, related concomitant medication, disease evaluation, progress follow-up, survival status, adverse events, laboratory evaluation, etc.

6.3 Description on Analysis Set

Full Analysis Set (FAS): it will include all patients who have been administrated with at least one dose of test drug.

6.4 Statistical Analysis Methods

All analyses will be conducted for FAS population. No statistical test will be conducted and all statistical analyses will be descriptive.

Efficacy analysis will be repeated according to the cohort defined by the number and category of previous treatment lines.

Descriptive statistical summary data will be used as appropriate. Continuous variables will be summarized by the number of observed values (n), average value, standard deviation (SD), median, quartile (Q1 and Q3), minimum, and maximum. Categorical variables will be summarized by frequency count and percentage for each category.

All statistical tests will give test statistics and their corresponding P values, and in Fisher's exact probability test, P values will be given directly. In the comparison between groups, it is considered that the difference tested is statistically significant when the P value is less than or equal to 0.05 by two-tailed test.

The difference analysis for qualitative data will be conducted by using chi-square test, Fisher exact probability test and Wilcoxon rank sum test. Quantitative data will be analyzed by t test, corrected t test, Wilcoxon rank sum test or covariance analysis.

Log-rank test will be used to compare survival time, and K-M method will be used to estimate median survival time.

6.4.1 Evaluation on Primary Endpoint

Objective Response Rate (ORR): It refers to the proportion of patients who have completed the 8-to-16-week treatment with afatinib before operation and have had a complete response or partial response (according to RECIST1.1) as confirmed by CT evaluation after 3 weeks in all patients who have completed the treatment. Only patients with measurable lesions at baseline will be analyzed.

6.4.2 Evaluation on Secondary Endpoints

Pathological downstaging rate: It is defined as the proportion of patients who have completed the 8-week treatment with afatinib before operation and have achieved a T stage downing of the tumor as confirmed by CT evaluation after 3 weeks in all patients who have completed the treatment.

Pathological complete response (pCR): It refers to the proportion of patients with no invasive carcinoma residual found in primary tumor lesions and lymph nodes under microscope by HE staining in all patients who have completed the treatment.

R0 resection rate: It is defined as the proportion of patients with negative surgical margin and no residual found under microscope after resection in all patients who have completed the treatment.

Event-free survival (EFS): It refers to the time from the first administration of afatinib in this study to any progression of disease precluding surgery, progression or recurrence of disease after surgery, progression of disease in the absence of surgery, or death from any cause (including any cause of death in the event of no progression) as recorded in CRF.

Progression-free survival (PFS): It refers to the time from the first administration of afatinib in this study to the disease progression or death (including any cause of death without disease progression) as recorded in CRF, regardless of whether the patient exits from the treatment or receives other anti-cancer treatment before progression. In the case of a patient who does not develop a progression or has died at the time of analysis, the latest evaluation date will be used for interpolation (censoring). (Or, if there is no tumor assessment after the baseline visit, the baseline visit date will be used).

Disease free survival (DFS): It refers to the time from radical surgery to relapse or death of a participant due to disease progression. In the case of a patient who still survives at the time of analysis, the latest evaluation date will be used for interpolation (censoring).

Overall survival (OS): It is defined as the time from random enrollment to death of participant due to any cause. In the case of a patient who still survives at the time of analysis, the date of last contact will be taken as the censoring date. In the event of a patient with the survival status unknown, the date when the patient is last known to be alive will be used for interpolation (censoring).

Health related quality of life (HRQol): The assessment is made according to the Quality of Life Scale for Lung Cancer Patients (EORTC-QLQ-C30 & LC13, Version 3).

6.4.3 Safety Analysis

The safety and tolerance evaluation of participants is based on the following analysis results:

Treatment-related adverse events

Treatment-related serious adverse events

Treatment-related adverse events that lead to discontinuation of medication with afatinib

All adverse events will be summarized according to MedDRA preferred terminology (PT) and CTCAE grade. A treatment-related adverse event refers to the adverse event that is determined by the investigator to be related to afatinib (or, a treatment-related adverse event refers to the adverse event that is determined by the investigator to be related to afatinib or platinum-doublet chemotherapy).

Time limit and scope of AE collection: all adverse events shall be collected 30 days after

the participant signs informed consent to the last administration of Afatinib [or platinum-based dual-drug chemotherapy]. From then on to the end of the study, only SAE related to Afatinib (or platinum-based dual-drug chemotherapy) needs to be collected.

7. Adverse Events

7.1Considerations/Precautions

Please refer to the detailed prescription information in the locally approved package of the related drugs or in the Investigator Brochure for research of afatinib for the potential adverse reactions caused by afatinib and/or platinum-doublet chemotherapy.

7.2Monitoring of Adverse Events

Adverse events of participants must be closely monitored, for example, through clinical related examinations. Assessment shall be made according to the importance, severity and relationship with test drug of adverse events.

The investigators shall be responsible for evaluating the relationship between all adverse events and the study drugs. The principal investigator can entrust other investigators participating in this study to make a judgment, but will still need to be responsible for it. The investigators must provide a list of qualified persons who accept the entrustment.

7.3 Definition of Adverse Event

7.3.1 Adverse Event

An adverse event refers to any adverse medical events that occur to the patients or participants who have received the treatment with study drugs. The adverse event is not necessarily causally related to the treatment. Therefore, the adverse event can be any adverse and meaningless sign (including abnormality in laboratory examination), symptom or disease temporarily associated with the study drugs used, regardless of whether the event is considered to be related to drugs.

The adverse events occurring in human body (whether related to drugs or not) include:

- Adverse events occurred during the use of drugs by professionals;
- Adverse events caused by overdose (intentionally or unintentionally);
- Adverse events caused by drug abuse;
- Adverse events caused by discontinuation of medication;
- The adverse event that may be caused solely by the patient's participation in the study (e.g. adverse events or serious adverse events due to discontinuation of antihypertensive drugs during elution period) must be reported as an adverse event even if it is not related to the study medication.

The event with no clinical pharmacological effect or with a clinical pharmacological effect that does not achieve the expected level, which has been recorded in the corresponding part of CRF, will not be taken as an adverse event.

7.3.2 Serious Adverse Event

A serious adverse event refers to any adverse medical event that meets one of the following conditions under any medication condition:

- Causing death;
- Life-threatening;
- Causing the patient to be hospitalized or prolonging the hospitalization time;
- Causing permanent or obvious loss of working ability or disability;
- Causing congenital malformation or congenital defect;
- Causing major medical events.

Life-threatening: The term "life threatening" is defined as "serious" and means that the participant is at the risk of death when such an adverse event occurs. It does not refer to any adverse event that may lead to death on condition that the situation gets worse.

Hospitalization: Any adverse event that results in hospitalization of a patient or prolongs the hospitalization time of patient will be considered serious unless one of the following conditions is met.

– The patient stays in hospital for observation for no more than 12 hours;

or

– The hospitalization is scheduled in advance (i.e. the surgery or elective surgery has been scheduled before commencement of this study);

or

– The hospitalization is not due to the adverse event (e.g. hospitalization for convalescent purposes).

Note: Any invasive treatment during hospitalization may meet the criteria of a "major medical event", so it may need to be reported as a serious adverse event according to clinical judgment. Moreover, if the local regulatory authority has proposed a stricter requirement in definition of such an event, the local regulations shall prevail.

Disability: It means that one's ability to engage in daily life is seriously impaired.

Major medical event: Any adverse event that may endanger the participants and may require intervention to prevent more serious cases can be considered as a serious adverse event. Please refer to the "World Health Organization Adverse Reaction Terms-Main Terminology" to determine a major medical event. These terms refer to or describe the status of a serious disease.

The reason why such an event shall be reported as a serious adverse event is that they may be related to a serious disease status, and special concern may be caused for the event reported as a SAE, which could promote necessary actions to be carried out.

7.3.3 Unexpected Adverse Event

An unexpected adverse event refers to any adverse drug-related event with the characteristics or severity inconsistent with criteria specified in the Investigator Brochure (or

in the instructions supplied with the marketed products). The report of unexpected adverse events is also made partially for the purpose of supplementing important information on the characteristics or severity of known and recorded adverse events. For example, the event with more speciality or severity compared with that described in the Investigator's Brochure shall be considered "unexpected". Specifically, for example, it may include: (a) acute renal failure that has been marked as an adverse event and followed by a complication of interstitial nephritis; and (b) the hepatitis reported initially as acute liver necrosis.

7.3.4 The Relationship between Adverse Events and Study Drugs

The evaluation on the relationship between an adverse event and study drugs is a comprehensive clinical judgment based on all the information obtained when completing the Case Report Form.

It could be evaluated as "Unrelated" under following conditions:

1. There are other clear explanations, such as traumatic hemorrhage at the surgical site;

or

2. It is considered as unreasonable, for example, a participant is hit by a car, but there is no evidence indicating that the drug-induced disorientation has caused the incident; or a cancer occurs only a few days after commencement of medication.

The affirmative result of the evaluation indicates that there are reasonable reasons to confirm the adverse events may be related to the study drugs.

Factors to be considered when evaluating the relationship between adverse events and study drugs include:

- Occuring in the short term after drug use: such an adverse event shall occur after drug administration. The time span between medication and occurrence of the event shall be considered in clinical evaluation of the event.
- The event disappears after discontinuation of medication (stimulation discontinued) and occurs again after resuming medication (stimulation resumed): when analyzing the clinical process of suspicious events, the reaction of the participant after discontinuation of medication (stimulation discontinued) or the reaction of the participant after resuming medication (stimulation resumed) shall be fully considered.
- Basic diseases, concomitant diseases and sporadic diseases: the evaluation shall be made to the natural course, treatment process and all other diseases that the patient may suffer from shall be made in each report;
- Concomitant medication or treatment: other drugs taken by the participant or other treatments received by the participant shall be checked to determine whether one of them is the cause of the adverse event;
- Known response patterns of certain a drug: clinical/preclinical;
- Pharmacology and pharmacokinetics of the study drugs: the pharmacokinetic characteristics (absorption, distribution, metabolism and excretion) of the study drugs shall

be considered in combination with the individual pharmacodynamic response of each participant.

7.3.5 Record of Adverse Events

All adverse events shall be collected from the signature of ICF by participants up to 30 days after the last administration. Then only SAE related to afatinib or platinum-doublet chemotherapy will need to be collected up to the completion of study.

In this study, disease progression will not be recorded as an AE, and death caused by disease progression will not be recorded as a SAE unless the investigators believe that the disease progression is related to afatinib (*or platinum-doublet chemotherapy*). Any new onset of cancer shall be recorded as a SAE, regardless of the time span between the medication of study drugs and the cancer occurrence.

Records must be supported by original data. Any abnormality in laboratory examination that is considered with clinical correlation (for example, those causing participants to withdraw from the study ahead of schedule, requiring treatment or causing obvious clinical manifestations, or those considered clinically relevant by investigators) shall be reported as an adverse event. Each event shall be described in detail, including the start and end dates, severity, relationship with the test drug, measures taken and outcome of the event.

7.4 Report of Serious Adverse Events/Pregnancy

All serious adverse events (SAEs) that meet the criteria of definition and occurr during the safety reporting period of this study, including laboratory examination abnormalities, must be reported to the personnel designated in the study documents immediately (within 24 hours after the investigators learn about the events). Any serious adverse event discovered by the investigators or working staff of the research center during the research process, whether it is related to the study drugs or not, must be recorded in the serious adverse event report form immediately after it has been known and reported to the ethics department of the research center and Boehringer Ingelheim within 24 hours. The serious adverse event related to the research treatment must be collected and reported, regardless of how long it has been since the last administration, even if the trial has been completed.

All serious adverse events shall be followed up until they are resolved or become stable, and an update report shall be submitted to the designated personnel. A simple level 4 laboratory examination abnormality (according to the CTCAE 4.0) will not be reported as a serious adverse reaction unless the investigators believe that the abnormality has met the standard proposed by the International Conference of Harmonization for serious adverse events (seeing the definition in section 7.3.2). The Level 4 laboratory examination abnormality that is found in the baseline period as the manifestion of diseases shall not be reported as a serious adverse event, though it meets the criteria in CTCAE 4.0, especially when the patient has been allowed

into the trial or not excluded from the trial. If there is a doubt about whether the abnormality shall be reported as a serious adverse event or not, the investigators may consult the research inspector. The laboratory examination abnormalities of level 4 as specified in CTCAE shall be recorded in the "Laboratory Information" page and checked regularly by medical inspectors. If it is uncertain whether an adverse event is caused by the disease research, it shall be reported as an AE or SAE.

If a female participant gets pregnant during the study, her medication of study drugs must be discontinued according to the instructions, and a report shall be sent immediately to the investigators. The investigators must report all pregnancies to the Sponsor and Boehringer Ingelheim within 24 hours, and shall also give the patients medical advice and discuss the risks of continuing pregnancy and the possible impact on the fetus. The patients shall be monitored until the pregnancy issue has been resolved. The pregnancies that occur within 90 days after the completion of study medication shall also be reported to the investigators. And the patients shall be monitored continuously until the pregnancy issue has been resolved.

7.5 Non-Serious Adverse Reactions to Drugs

A non-serious adverse reaction refers to the adverse event related to treatment but not meeting the severity criteria as evaluated by the investigators. The severity criteria are detailed in section 7.3.2 of the protocol.

The non-serious adverse reactions that occur during the safety reporting period of this study must be reported to Boehringer Ingelheim within 7 natural days after being learnt.

8. Data Processing and Preservation

8.1 Case Report Form (CRF)

A Case Report Form (CRF) must be completed and signed by the principal investigator or the representative authorized by the principal investigator for each patient selected. This also applies to the recording of CRF for patients who fail to complete the trial (even during the screening period prior to randomization, provided that the case report form has been filled in). In the event that a patient withdraws from the study treatment, the reason for withdrawal must be recorded on CRF. If the patient withdraws from the study due to treatment-limited adverse events, comprehensive efforts shall be made to clearly record the results.

All forms shall be printed or recorded with indelible ink and must be legible. The errors can be crossed out instead of being removed, and the revised contents can be inserted accordingly. The investigators or their authorized representatives shall sign and date the revisions.

8.2 Preservation of Research Documents, Case Report Forms and Records

8.2.1 Preservation of Investigators' Folders/Files

The investigators must keep comprehensive and accurate records during the implementation of the research, in order to ensure that the implementation of the research is

fully recorded and the research data can be verified subsequently. These documents should be divided into two different types:

(1) Research documents of the investigators;

(2) Original clinical records of the patients.

The research documents of investigators include the trial protocol and revisions, approvals from the Independent Ethics Committee/Institutional Review Committee and the government, ICF sample, drug records, personnel resumes, power of attorney and other relevant documents/correspondence, etc.

Original clinical records of the patients (generally referring to the key effectiveness/safety parameters, defined before the start of the project and recorded outside the medical record form) usually include patient hospitalization/outpatient records, doctors' and nurses' records, appointment forms, original laboratory reports, electrocardiogram, electroencephalogram, X-ray, pathology and special evaluation reports, signed informed consent forms, consultation letters, patient screening and recruitment forms.

8.3 Archiving

The data to be entered into the CRF must be consistent with the original documents, or they may be recorded directly into the CRF. In the case of directly recording into CRF, the recorded content will be taken as the original data. The parameters of the original data must be verified and the information of the data source must be recorded. The research documents and all the original materials shall be kept until the notification for destruction from the Sponsor is received.

8.4 Establishment of Database

After receiving the CRFs, the statisticians will propose the questions to the investigators for verification through the clinical inspector, and the investigators shall make a response as soon as possible. The statisticians shall establish the database in time. After the database is verified, the data will be locked by the principal investigator, Sponsor, statisticians and clinical inspectors. In consideration of data safety, the unauthorized person cannot access and modify the data, and the data must be backed up.

9. References:

1. Bray F, Ferlay J, Soerjomataram I, et al. Global cancer statistics 2018: GLOBOCAN estimates of incidence and mortality worldwide for 36 cancers in 185 countries. CA Cancer J Clin. 2018.

2. Chen Wanqing, Zhang Siwei, Zheng Rongshou, et al. Analysis of Cancer Onset and Death Cases in China’s Cancer Registration Region in 2007. China Cancer. 2011;20(03):162-9.

3. Duan Jijun, Yan Yaqiong, Yang Niannian, et al. International Comparative Analysis of Malignant Cancer Onset and Death Cases in China. Chinese Journal of the Frontiers of Medical Science (Electronic Version). 2016;8(07):17-23.

4. Chen Wanqing, Zheng Rongshou, Baade Peter D, et al. Cancer statistics in China, 2015.

CA: a cancer journal for clinicians,2016,66(2).

5. www.seer.cancer.gov. Accessed 06/24/2019

6. Herbst RS, Heymach JV, Lippman SM. Lung cancer. New England Journal of Medicine, 2008, 359(13): 1367-1380

7. Rami-Porta R, Asamura H, Travis WD, et al. Lung cancer - major changes in the American Joint Committee on Cancer eighth edition cancer staging manual. CA Cancer J Clin. 2017; 67(2): 138-55.

8. Goldstraw P, Chansky K, Crowley J, et al. The IASLC Lung Cancer Staging Project: Proposals for Revision of the TNM Stage Groupings in the Forthcoming (Eighth) Edition of the TNM Classification for Lung Cancer. J Thorac Oncol. 2016;11(1):39-51.

9. Coate LE, John TTsao MS. Molecular predictive and prognostic markers in non-small-cell lung cancer. Lancet Oncology, 2009, 10(10): 1001-1010.

10. Pignon JP, Tribodet H, Scagliotti GV, et al. Lung adjuvant cisplatin evaluation: a pooled analysis by the LACE Collaborative Group. J Clin Oncol. 2008;26(21):3552-9.

11. Cancer Genome Atlas Research N. Comprehensive genomic characterization of squamous cell lung cancers. Nature. 2012;489(7417):519-25.

12. Cancer Genome Atlas Research Network. Comprehensive molecular profiling of lung adenocarcinoma. Nature. 2014;511(7511):543-50.

13. Cancer Genome Atlas Research Network. Comprehensive genomic characterization of squamous cell lung cancers.Nature,2012, 489 (7417): 519-525.

14. Cancer Genome Atlas Research Network. Comprehensive molecular profiling of lung adenocarcinoma.Nature,2014,511(7511):543-550.

15. George J,Lim JS,Jang SJ,et al. Comprehensive genomic profiles of small cell lung cancer.Nature,2015,524(7563):47-53.

16. Weinstein IB. Cancer. Addition to oncogenes-the Achilles heal of Cancer. Science, 2002,297(5578):63-64

17. Cancer Genome Atlas Research Network. Comprehensive molecular profiling of lung adenocarcinoma.Nature,2014,511(7511):543-550

18. Fernandez-Cuesta L, Plenker D, Osada H, et al. CD74-NRG1 fusions in lung adenocarcinoma.Cancer Discov,2014,4 (4):415-422

19. Nakaoku T, Tsuta K, Ichikawa H, et al. Druggable oncogene fusions in invasive mucinous lung adenocarcinoma. Clin Cancer Res,2014,20(12):3087-3093.

20. Kim Y, Hammerman PS, Kim J, et al. Integrative and comparative genomic analysis of lung squamous cell carcinomas in East Asian patients.J Clin Oncol,2014,32(2):121-128.

21. Yarden Y, Sliwkowski MX. Untangling the ErbB signaling network. Nat. Rev. Mol. Cell Biol.2001.2:127–37

22. De Luca A, Carotenuto A, Rachiglio A, Gallo M, Maiello MR, et al. The role of the EGFR signaling in tumor microenvironment. J. Cell Physiol.2008. 214:559–67

23. Jimeno A, Hidalgo M. Pharmacogenomics of epidermal growth factor receptor (EGFR) tyrosine kinase inhibitors. Biochim. Biophys. 2006. 1766:217–29

24. Mosesson Y, Yarden Y. Oncogenic growth factor receptors: implications for signal transduction therapy. Semin. Cancer Biol. 2004.14:262–70

25. Ciardiello F, Tortora G. EGFR antagonists in cancer treatment. N. Engl. J. Med. 2008.358:1160–74

26. Woodburn JR.. The epidermal growth factor receptor and its inhibition in cancer therapy.

Pharmacol. Ther.1999. 82:241–50

27. Herbst RS, Bunn PA Jr. Targeting the epidermal growth factor receptor in nonsmall cell lung

cancer. Clin. Cancer Res.2003. 9:5813–24

28. Hazan RB, Norton L.The epidermal growth factor receptor modulates the interaction of Ecadherin with the actin cytoskeleton. J Biol. Chem. 1998.273:9078–84

29. Ellerbroek SM, Halbleib JM, Benavidez M, Warmka JK, Wattenberg EV, et al. Phosphatidylinositol 3–kinase activity in epidermal growth factor–stimulated matrix metalloproteinase–9 production and cell surface association. Cancer Res. 2001.61:1855–61

30. Lee SY, Kim MJ, Jin G,et al. Somatic mutations in epidermal growth factor receptor signaling pathway genes in non-small cell lung cancers. J Thorac Oncol, 2010,11(11):1734-1740.

31. Yun CH, Boggon TJ, Li Y, Woo MS, et al. Structures of lung cancer–derived EGFR mutants and inhibitor complexes: mechanism of activation and insights into differential inhibitor sensitivity. Cancer Cell. 2007.11:217–27

32. Kumar A, Petri ET, Halmos B, Boggon TJ. Structure and clinical relevance of the epidermal growth factor receptor in human cancer. J. Clin. Oncol. 2008.26:1742–51

33. Park K，Tan EH，O’Byme K，et a1．Afatinib versus gefitinib as firsIline treatment of patients with EGFR mutation-positive non-small-cell lung cancer(LUX-Lung 7): a phase 2B，open-1abel, randomized controlled trial．Lancet 0ncol,2016,17(5);577-589．

34. NSCLC Meta-analyses Collaborative Group. Preoperative chemotherapy for non-small-cell lung cancer: a systematic review and meta-analysis of individual participant data. The Lancet,2014,383(9928)

35. Zhong WZ, Wang Q, Mao WM, et al. Gefitinib versus vinorelbine plus cisplatin as adjuvant treatment for stage II-IIIA (N1-N2) EGFR-mutant NSCLC (ADJUVANT/CTONG1104): a randomised, open-label, phase 3 study. Lancet Oncol. 2018;19(1):139-48

36. Li,N.,Ou,W.,Ye,X., et al.Pemetrexed-carboplatin adjuvant chemotherapy with or without gefitinib in resected stage IIIA-N2 non-small cell lung cancer harbouring EGFR mutations: A randomized, phase II study.Annals of surgical oncology,2014,21(6):2091-2096.

37. Kappers I, Klomp HM, Burgers JA, et al. Neoadjuvant (induction) erlotinib response in stage IIIA non-small-cell lung cancer. J Clin Oncol. 2008;26(25):4205-7.

38. Wang Q, Wang H, Li P, et al. Erlotinib-based perioperative adjuvant therapy for a case of unresectable stage IIIA (N2) nonsmall cell lung cancer. Am J Med Sci. 2010;340(4):321-5

39. Zhong WZ, Chen KN, Chen C, et al. Erlotinib Versus Gemcitabine Plus Cisplatin as Neoadjuvant Treatment of Stage IIIA-N2 EGFR-Mutant Non-Small-Cell Lung Cancer (EMERGING-CTONG 1103): A Randomized Phase II Study. Journal of clinical oncology 2019:Jco1900075.

40. Sequist L V, et al.A phase II study of neoadjuvant afatinib, chemoradiation and surgery for stage III EGFR mutation-positive NSCLC. J Clin Oncol. 2018: 8544-8544.

41. Chow S, Shao J, Wang H. 2008. Sample Size Calculations in Clinical Research. 2nd Ed. Chapman & Hall/CRC Biostatistics Series. page 85.

10. Appendixes

Appendix I ECOG Scoring Criteria

Score	Performance Status
0	Fully active, able to carry on all pre-disease performance without restriction (Karnofsky 90-100 points)

1	Restricted in physically strenuous activity but ambulatory and able to carry out work of a light or sedentary nature, e.g., light house work, office work (Karnofsky 70-80 points)
2	Ambulatory and capable of all selfcare but unable to carry out any work activities. Up and about more than 50% of waking hours (Karnofsky 50-60 points)
3	Capable of only limited selfcare, confined to bed or chair more than 50% of waking hours (Karnofsky 30-40 points)
4	Completely disabled. Cannot carry on any selfcare. Totally confined to bed or chair (Karnofsky 10-20 points)

Appendix II TNM Classification – International Lung Cancer Staging Standard (8th Edition)

**Primary lesions (T staging)**	
**Tx**	Size of primary tumor immeasurable; cancer cell in sputum/bronchial washings but tumor not be assessed in imaging or bronchoscopy.
**T0**	No evidence of tumor
**Tis**	Carcinoma in situ
**T1**	Primary tumors≤3 cm surrounded by lung/visceral pleura, not involving main bronchus (some rare superficial tumors limited to bronchial wall are classified as T1 regardless of size even if they involve the part above the main bronchus.)
**T1a mi**	Minimally invasive carcinoma
**T1a ss**	Superficial spreading tumor of any size, but limited in the airway wall or main bronchus
**T1a**	Maximum diameter of tumor ≤1cm
**T1b**	1cm<maximum diameter of tumor≤2cm
**T1c**	2cm<maximum diameter of tumor≤3cm
**T2**	3cm<maximum diameter of tumor≤5cm， or involvement of main bronchus without carina (distance from carina≥2cm), or invasion visceral pleural, or atelectasis or post obstructive pneumonitis extending to hilum but not the whole lung.
**T2 Visc Pl**	invasion of visceral pleural *
**T2 Centr**	Invasion of main bronchus (excluding carina); obstructive pneumonia, partial or complete atelectasis *.
**T2a**	3cm<maximum diameter of tumor≤4cm.
**T2b**	4cm<maximum diameter of tumor≤5cm.
	*In the case of of 3cm<maximum diameter of tumor≤4cm, T2a； in the case of 4cm<maximum diameter of tumor≤5cm, T2b.
**T3**	5cm<maximum diameter of tumor≤7cm, or directly invade one of the following: chest wall (including superior sulcus tumor of lung); diaphragm; phrenic nerve; mediastinal pleura; pericardium; or the tumor is located in

	the main bronchus <2 cm away from the carina, but the carina is not involved; causing atelectasis or obstructive pneumonia; satellite nodules appear in the same lobe of the primary tumor.
**T3**	5cm<maximum diameter of tumor≤7cm
**T3 Inv**	Direct invasion of chest wall, phrenic nerve and pericardium.
**T3 Satell**	Solitary cancer nodule appears in the same lobe.
**T4**	7 cm < maximum diameter of tumor; tumors of any size invade any of the following organs, including mediastinum, heart, large blood vessels, carina, recurrent laryngeal nerve, main trachea, esophagus, vertebral body and diaphragm; solitary cancer nodules in different lung lobes on the same side.
**T4**	7cm<maximum diameter of tumor
**T4 Inv**	Regardless of size, invasion of specific organs.
**T4 Ipsi Nod**	Solitary cancer nodules in different lung lobes on the same side.
Regional lymphnode (N staging)	
**Nx**	Regional lymphnodes cannot be evaluated.
**No**	No regional lymph node metastasis.
**N1**	Metastasis to ipsilateral peribronchial lymphnodes and/or ipsilateral hilar lymphnodes, and primary tumor directly involving pulmonary lymphnodes.
**N2**	Metastasis to ipsilateral mediastinum and/or subtrochanteric lymph nodes.
**N3**	Metastasis to contralateral mediastinal lymphnodes, contralateral hilar lymphnodes, ipsilateral or contralateral scalene muscle lymphnodes, or supraclavicular lymphnodes.
Distant metastasis (M staging)	
**Mx**	It is impossible to evaluate whether there is any distant metastasis.
**M0**	No distant metastasis.

**M1**	Distant metastasis.		
**M1a Pl Dissem**	Pleural dissemination (malignant pleural effusion, pericardial effusion or pleural nodule), pleural effusion of lung cancer is caused by tumor, and pleural effusion of a few patients is negative in multiple cytological examinations, neither bloody nor exudative. If it is believed that exudation has nothing to do with tumor based on various factors and clinical judgment, pleural effusion should not be included in staging factors.		
**M1a Contr Nod**	Solitary cancer nodules appear in the contralateral lobe.		
**M1b**	Distant single-organ metastasis.		
**M1c**	Distant single-organ metastasis or multiple-organ metastases.		
**Clinical stage**			
**Delitescence stage**	TX	N0	M0
**Stage 0**	Tis	N0	M0
**StageI A1**	T1a(mi)	N0	M0
	T1a	N0	M0
**Stage I A2**	T1b	N0	M0
**Stage I A3**	T1c	N0	M0
**Stage I B**	T2a	N0	M0
**Stage II A**	T2b	N0	M0
**Stage II B**	T1a-c	N1	M0
	T2a	N1	M0
	T2b	N1	M0
**Stage III A**	T1a-c	N2	M0
	T2a-b	N2	M0
	T3	N1	M0

	T4	N0	M0
	T4	N1	M0
**Stage III B**	T1a-c	N3	M0
	T2a-b	N3	M0
	T3	N2	M0
	T4	N2	M0
**Stage III C**	T3	N3	M0
	T4	N3	M0
**Stage IV A**	Any T	Any N	M1a
	Any T	Any N	M1b
**Stage IV B**	Any T	Any N	M1c

Appendix III NYHAFunctional Classification

	**HYHA Functional Classification**
**Class I**	Patients with cardiac disease but resulting in no limitation of physical activity. Ordinary physical activity does not cause undue fatigue, palpitation, dyspnea or anginal pain.
**Class II**	Patients with cardiac disease resulting slight limitation of physical activity. They are comfortable at rest. Ordinary physical activity results fatigue, palpitation, dyspnea or anginal pain.
**Class III**	Patients with cardiac disease resulting in marked limitation of physical activity. There are comfortable at rest. Less than ordinary activity causes fatigue, palpitation, dyspnea or anginal pain.
**Class IV**	Patients with cardiac disease resulting in inability to carry on any physical activity without discomfort. Symptoms of heart failure or the anginal syndrome may be present even at rest. If any physical activity is undertaken, discomfort increases.

Appendix IV Common Terminology Criteria for Adverse Events of NCI (CTCAE v4.0)

Blood/Marrow						
		Grade				
Adverse Event	Short Name	1	2	3	4	5
Bone marrow hypocellular	Bone marrow hypocellular	Mildly hypocellular or <=25% reduction from normal cellularity for age	Moderately hypocellular or >25-<=50% reduction from normal cellularity for age	Severely hypocellular or >50-<=75% reduction cellularity from normal for age	—	Death
CD4 Cell Count	CD4 Cell Count	<LLN-500/mm^3<br><LLN-0.5 ×10^9/L	<500-200/ mm^3<br><0.5-0.2×10^9/L	<200-50/mm^3<br><0.2×0.05-10^9/L	<50/mm^3<br><0.05×10^9/L	Death
Haptoglobin	Haptoglobin	<LLN	—	None	—	Death
hemoglobin	hemoglobin	<LLN-10.0g/dL<br><LLN-6.2mmol/L<br><LLN-100g/L	<10.0-8.0g/dL<br><6.2-4.9mmol/L<br><100-80g/L	<8.0-6.5g/dL<br><4.9-4.0mmol/L<br><80-65g/L	<6.5g/dL<br><4.0mmol/L<br><65g/L	Death
Hemolysis (e.g. immune hemolytic anemia, drug-induced hemolysis)	Hemolysis	Laboratory evidence of hemolysis only (e.g., direct antiglobulin test; DAT; Coombs'; schistocytes; decreased haptoglobin)	Evidence of hemolysis and >=2 gm decrease in hemoglobin.	Transfusion or medical intervention indicated (e.g., steroids)	Consequences caused by hemolysis (e.g. renal failure, hypotension, bronchospasm, emergency splenectomy)	Death
White blood cells (total WBC)	White blood cells	<LLN-3000/mm^3<br><LLN-3.0×10^9/L	<3000-2000/mm^3<br><3.0-2.0×10^9/L	<2000-1000/mm^3<br><2.0-1.0×10^9/L	<1000/mm^3<br><1.0×10^9/L	Death
Lymphocytopenia	Lymphocytopenia	<LLN-800/mm^3<br><LLN×0.8-10^9/L	<800-500/mm^3<br><0.8-0.5×10^9/L	<500-200/mm^3<br><0.5-0.2×10^9/L	<200/mm^3<br><0.2×10^9/L	Death
Myelodysplasia	Myelodysplasia	—	—	Genetic variation of bone marrow cells (bone marrow bud cells < 5%)	RAEB or RAEB-T (bone marrow bud cells <5%)	Death

Neutrophil / granular cells (ANC/AGC)	Neutrophil	<LLN-1500/mm^3<br><LLN-1.5×10^9/L	<1500-1000/mm^3<br><1.5-1.0×10^9/L	<1000-500/mm^3<br><1.0-0.5×10^9/L	<500/mm^3<br><0.5×10^9/L	Death
Platelet	Platelet	<LLN-75,000/mm^3<br><LLN-75.0×10^9/L	<75,000-50,000/mm^3<br><75.0-50.0×10^9/L	<50,000-25,000/mm^3<br><50.0-25.0×10^9/L	<25,000/mm^3<br><25.0×10^9/L	Death
Splenic function	Splenic function	Incidental findings (e.g., HowellJolly bodies)	Prophylactic antibiotics indicated	—	Life-threatening	Death
Blood/bone marrow-other situations (specify, —)	Blood-other situations (specify)	Mild	Moderate	Severe	Life-threatening; disabling	Death

Arrhythmia						
		Grade				
Adverse Event	Short Name	1	2	3	4	5
Conduction abnormality/atrioventricular heart block – select	Conduction abnormality – select	Asymptomic, intervention not indicated	Non-urgent medical intervention indicated	Incompletely controlled medically or controlled with device (e.g., pacemaker)	Life-threatening (e.g., arrhythmia associated with CHF, hypotension, syncope, shock)	Death
Palpitations	Palpitations	Present	With associated symptoms (e.g., lightheadedness, shortness of breath)	—	—	—
Cardiac Arrhythmia – Other	Cardiac Arrhythmia	Mild	Moderate	Severe	Life-threatening; disabling	Death
Cardiac						
		Grade				
Adverse Event	Short Name	1	2	3	4	5
Cardiac ischemia/infarction	Cardiac ischemia/infarction	Asymptomatic arterial narrowing without ischemia	Asymptomatic and testing suggesting ischemia; stable angina	Symptomatic and testing consistent with ischemia; unstable angina; intervention indicated	Acute myocardial infarction	Death
Cardiac troponin T	cTnT	0.03 – <0.05 ng/mL	0.05 – <0.1 ng/mL	0.1 – <0.2 ng/mL	0.2 ng/mL	—
Hypertension	Hypertension	Asymptomatic, transient (<24 hrs) increase by >20 mmHg (diastolic) or to >150/100 if previously WNL; intervention not indicated	Recurrent or persistent (≥24 hrs) or symptomatic increase by >20 mmHg (diastolic) or to >150/100 mmHg from normal blood pressure at above condition; monotherapy may be indicated	Requiring more than one drug or more intensive therapy than previously	Life-threatening consequences (e.g., hypertensive crisis)	Death
Remark: There are different grading standards for pediatric patients, which are not involved in this study and are not listed here.						

General						
		Grade				
Adverse Event	Short Name	1	2	3	4	5
Fatigue (lethargy, malaise,asthenia)	Fatigue	Mild fatigue over baseline	Moderate or causing difficulty performing some ADL	Severe fatigue interfering with ADL	Disabling	—
Fever (in the absence of neutropenia, where neutropenia is defined as ANC <1.0 x 10 ^{9}/L)	Fever	38.0-39.0°C (100.4-102.2°F)	>39.0-40.0°C (102.3-104.0°F)	>40.0°C (>104.0°F) ≤24hrs	>40.0°C (>104.0°F) >24hrs	Death
Hypothermia	Hypothermi a	—	35->32°C 95->89.6°F	32->28°C 89.6->82.4°F	≤28 °C 82.4°F or life-threatening consequences (e.g., coma, hypotension, pulmonary edema, acidemia, ventricular fibrillation)	Death
Insomnia	Insomnia	Occasional difficulty sleeping, not interfering with function	Difficulty sleeping, interfering with function but not interfering with ADL	Frequent difficulty sleeping, interfering with ADL	Disabling	—
REMARK: If pain or other symptoms interfere with sleep, do NOT grade as insomnia. Grade primary event(s) causing insomnia.						
Rigors/chills	Rigors/chills	Mild	Moderate, narcotics indicated	Severe or prolonged, not responsive to narcotics	—	—
Sweating (diaphoresis)	Sweating	Mild and occasional	Frequent or drenching	—	—	—
Weight gain	Weight gain	5 – <10% from baseline	10 – <20% from baseline	≥20% from baseline	—	Death
Weight loss	Weight loss	5 to <10% from baseline; intervention not indicated	10 – <20% from baseline; nutritional support indicated	≥20% from baseline; tube feeding or TPN indicated	—	—
General Symptoms – Other (Specify, __)	General Symptoms – Other (Specify)	Mild	Moderate	Severe	Life-threatening; disabling	Death

Gastrointestinal						
		Grade				
Adverse Event	Short Name	1	2	3	4	5
NOTE: Abdominal pain or cramping is graded as Pain – Select in the PAIN CATEGORY.						
Anorexia	Anorexia	Loss of appetite without alteration in eating habits	Oral intake altered without significant weight loss or malnutrition; oral nutritional supplements indicated	Associated with significant weight loss or malnutrition (e.g., inadequate oral caloric and/or fluid intake); IV fluids, tube feedings or TPN indicated	Life-threatening	Death
Constipation	Constipation	Occasional or intermittent symptoms; occasional use of stool softeners, laxatives, dietary modification, or enema	Persistent symptoms with regular use of laxatives or enemas indicated	Symptoms interfering with ADL; obstipation with manual evacuation indicated	Life-threatening (e.g., obstruction, toxic megacolon	Death
ALSO CONSIDER: Ileus, GI (functional obstruction of bowel, i.e., neuroconstipation); Obstruction, GI – Select.						
Dehydration	Dehydration	Increased oral fluids indicated; dry mucous membranes; diminished skin turgor	IV fluids indicated <24 hrs	IV fluids indicated ≥ 24hrs	Life-threatening (e.g., hemodynamic collapse)	Death
ALSO CONSIDER: Diarrhea; Hypotension; Vomiting.						
Diarrhea	Diarrhea	Increase of <4 stools per day over baseline; mild increase in ostomy output compared to baseline	Increase of 4 – 6 stools per day over baseline; IV fluids indicated <24hrs; moderate increase in ostomy output compared to baseline; not interfering with ADL	Increase of ≥7 stools per day over baseline; incontinence; IV fluids ≥24 hrs; hospitalization; severe increase in ostomy output compared to baseline; interfering with ADL	Life-threatening (e.g., hemodynamic collapse)	Death
REMARK: Diarrhea includes diarrhea of small bowel or colonic origin, and/or ostomy diarrhea.<br>ALSO CONSIDER: Dehydration; Hypotension.						
Distension	Distension	Asymptomatic	Symptomatic, but not interfering with GI function	Symptomatic, interfering with GI function	—	Death
ALSO CONSIDER: Ascites (non-malignant); Ileus, GI (functional obstruction of bowel, i.e., neuroconstipation); Obstruction, GI – Select.						
Flatulence	Flatulence	Mild	Moderate	—	—	—
Gastritis (including bile reflux gastritis)	Gastritis	Asymptomatic, radiographic or endoscopic findings only	Symptomatic; altered gastric function (e.g., inadequate oral caloric or fluid intake); IV fluids indicated <24 hrs	Symptomatic and severely altered gastric function (e.g., inadequate oral caloric or fluid intake); IV fluids, tube feedings, or TPN indicated ≥24 hrs	Life-threatening ; operative intervention requiring complete organ resection (e.g., gastrectomy)	Death
ALSO CONSIDER: Hemorrhage, GI – Select; Ulcer, GI – Select.						

NOTE: Head and neck soft tissue necrosis is graded as Soft tissue necrosis – Select in the MUSCULOSKELETAL/SOFT TISSUE CATEGORY.						
Heartburn/dyspepsia	Heartburn	Mild	Moderate	Severe	—	—
Mucositis/stomatitis (functional/symptomatic)	Mucositis (functional/ symptomatic) – Select	Upper aerodigestive tract sites: Minimal symptoms, normal diet; minimal respiratory symptoms but not interfering with function	Upper aerodigestive tract sites: Symptomatic but can eat and swallow modified diet; respiratory symptoms interfering with function but not interfering with ADL	Upper aerodigestive tract sites: Symptomatic and unable to adequately aliment or hydrate orally; respiratory symptoms interfering with ADL	Symptoms associated with life-threatening consequences	Death
Nausea	Nausea	Loss of appetite without alteration in eating habits	Oral intake decreased without significant weight loss, dehydration or malnutrition; IV fluids indicated <24 hrs	Inadequate oral caloric or fluid intake; IV fluids, tube feedings, or TPN indicated ≥24 hrs	Life-threatening consequences	Death
Vomiting	Vomiting	1 episode in 24 hrs	2 – 5 episodes in 24 hrs; IV fluids indicated <24 hrs	≥6 episodes in 24 hrs; IV fluids, or TPN indicated ≥24 hrs	Life-threatening	Death
Hepatic						
Alkaline phosphatase	WNL	>ULN ~ 2.5×ULN	>2.5×ULN ~ 5.0×ULN	>5.0×ULN ~ 20.0×ULN	>20.0×ULN	
Bilirubin	WNL	>ULN ~ 1.5×ULN	>1.5×ULN ~ 3.0×ULN	>3.0×ULN ~ 10.0×ULN	>10.0×ULN	
GGT (γ-Glutamyl transpeptidase)	WNL	>ULN ~ 2.5×ULN	>2.5×ULN ~ 5.0×ULN	>5.0×ULN ~ 20.0×ULN	>20.0×ULN	
Liver	Absent	-	-	Present	-	

enlargement	Note: Grading should only be performed when liver enlargement is caused by treatment-related adverse reactions, including venous occlusion diseases.				
Hypoalbuminemia	WNL	<LLN ~ 3 g/dL	≥2 ~ <3 g/dL	<2 g/dL	-
Liver dysfunction/ Liver failure (clinical)	Normal	-	-	Asterixis	Encephalopathy or coma
Portal vein blood flow	Normal	-	Decreased	Portal vein countercurrent	-
SGOT (AST) (serum glutamic oxaloacetic transaminase)	WNL	>ULN ~ 2.5×ULN	>2.5×ULN ~ 5.0×ULN	>5.0×ULN ~ 20.0×ULN	>20.0×ULN
SGPT (ALT) (serum glutamic pyruvic transaminase)	WNL	>ULN ~ 2.5×ULN	>2.5×ULN ~ 5.0×ULN	>5.0×ULN ~ 20.0×ULN	>20.0×ULN
Hepatic – Other (Specify, ___)	Absent	Mild	Moderate	Severe	Life-threatening or disabling

Neurology						
		Grade				
Adverse Event	Short Name	1	2	3	4	5
Apnea	Apnea	—	—	Present	Intubation indicated	Death
ALSO CONSIDER: Fever (in the absence of neutropenia, where neutropenia is defined as ANC <1.0 x 10 9/L); Infection – Select; Pain – Select; Vomiting						
Ataxia (incoordination)	Ataxia	Asymptomatic	Symptomatic, not interfering interfering with ADL	Symptomatic, interfering with ADL; mechanical assistance indicated	Disabling	Death
REMARK: Ataxia (incoordination) refers to the consequence of medical or operative intervention.						
Dizziness	Dizziness	With head movements or nystagmus only; not interfering with function	Interfering with function, but not interfering with ADL	Interfering with ADL	Disabling	Death
Memory impairment	Memory impairment	Memory impairment not interfering with function	Memory impairment interfering with function, but not interfering with ADL	Memory impairment interfering with ADL	Amnesia	—
Mood alteration —Select: —Agitation —Anxiety —Depression	Mood alteration —Select:	Mild mood alteration not interfering with function	Moderate mood alteration interfering with function, but not interfering with ADL; medication indicated	Severe mood alteration interfering with ADL	Suicidal ideation; danger to self or others	Death
Personality/behavioral	Personality	Change, but not adversely affecting patient or family	Change, adversely affecting patient or family	Mental health intervention indicated	Change harmful to others or self; hospitalization indicated	Death
Psychosis (hallucinations/ delusions)	Psychosis	—	Transient episode	Interfering with ADL; medication, supervision or restraints indicated	Harmful to others or self; life-threatening	Death
Neuropathy: sensory	Neuropathy-sensory	Asymptomatic; loss of deep tendon reflexes or paresthesia (including tingling) but not interfering with functio	Sensory alteration or paresthesia (including tingling), interfering with function, but not interfering with ADL	Sensory alteration or paresthesia interfering with ADL	Disabling	Death
Somnolence/depressed level of consciousness	Somnolence	—	Somnolence or sedation interfering with function, but not interfering with ADL	Obtundation or stupor; difficult to arouse; interfering with ADL	Coma	Death

Pain						
		Grade				
Adverse Event	Short Name	1	2	3	4	5
Pain – Select: 'Select ' AEs appear at the end of the CATEGORY.	Pain – Select:	Mild pain not interfering with function	Moderate pain; pain or analgesics interfering with function, but not interfering with ADL	Severe pain; pain or analgesics severely interfering with ADL	Disabling	Death
Pain – Other (Specify, __)	Pain – Other (Specify)	Mild pain not interfering with function	Moderate pain; pain or analgesics interfering with function, but not interfering	Severe pain; pain or analgesics severely interfering with ADL	Disabling	Death

			with ADL			

Hemorrhage/Bleeding						
Coagulation-related						
		Grade				
Adverse Event	Short Name	1	2	3	4	5
Human Fibrinogen	Human Fibrinogen	<1.0 – 0.75 x LLN or <25% decrease from baseline	<0.75 – 0.5 x LLN or 25 – <50% decrease from baseline	<0.5 – 0.25 x LLN or 50 – <75% decrease from baseline	<0.25 x LLN or 75% decrease from baseline or absolute value <50 mg/dL	Death
INR	INR	>1-1.5×ULN	>1.5-2×ULN	>2×ULN	—	—
PTT	PTT	>1-1.5×ULN	>1.5-2×ULN	>2×ULN	—	—
Adverse Event	Short Name	1	2	3	4	5
Hemorrhage/Bleeding	Hemorrhage	Mild without transfusion	—	Transfusion indicated	Massive bleeding, requiring major intervention	Death

Appendix V EORTC QLQ-C30 (version 3) Life Quality Questionnaires

We are interested in some things about you and your health. Please answer all of the questions yourself by circling the number that best applies to you. There are no "right" or "wrong" answers. The information that you provide will remain strictly confidential.

Please fill in your first initial:______________________

Your birth date (Day, Month, Year):_________________

Today's date(Day, Month, Year):____________________

During the past week:	Not at all	A Little	Quite a Bit	Very Much
1. Do you have any trouble doing strenuous activities, like carrying a heavy shopping bag or a suitcase?	1	2	3	4
2. Do you have any trouble taking a long walk?	1	2	3	4
3. Do you have any trouble taking a short walk outside of the house?	1	2	3	4
4. Do you need to stay in bed or a chair during the day?	1	2	3	4
5. Do you need help with eating, dressing, washing yourself or using the toilet?	1	2	3	4
**During the past week:**	**Not at All**	**A Little**	**Quite a Bit**	**Very Much**
6. Were you limited in doing either your work or other daily activities?	1	2	3	4
7. Were you limited in pursuing your hobbies or other leisure time activities?	1	2	3	4
8. Were you short of breath?	1	2	3	4
9. Have you had pain?	1	2	3	4
10. Did you need to rest?	1	2	3	4
11.Have you had trouble sleeping?	1	2	3	4
12. Have you felt weak?	1	2	3	4
13. Have you lacked appetite?	1	2	3	4

14. Have you felt nauseated?	1	2	3	4
15. Have you vomited?	1	2	3	4
16. Have you been constipated?	1	2	3	4
**During the past week:**	**Not at All**	**A Little**	**Quite a Bit**	**Very Much**
17. Have you had diarrhea?	1	2	3	4
18. Were you tired?	1	2	3	4
19. Did pain interfere with your daily activities?	1	2	3	4
20. Have you had difficulty in concentrating on things, like reading a newspaper or watch television?	1	2	3	4
21. Did you feel tense?	1	2	3	4
22. Did you worry?	1	2	3	4
23. Did you feel irritable?	1	2	3	4
24. Did you feel depressed?	1	2	3	4
25. Have you had difficulty remembering things?	1	2	3	4
26. Has your physical condition or medical treatment interfered with your family life?	1	2	3	4
27. Has your physical condition or medical treatment interfered with your social activities?	1	2	3	4
28. Has your physical condition or medical treatment caused you financial difficulties?	1	2	3	4

For the following questions please circle the number between 1 and 7 that best to you, numbers 1-7 represent Grades from "very poor" to "Excellent", respectively :

29. How would you rate your overall health during the past week?

1	2	3	4	5	6	7
Very poor						Excellent

30. How would you rate your overall quality of life during the past week?

1	2	3	4	5	6	7
Very poor						Excellent

EORTC QLQ-LC13 Life Quality Questionnaires

Patients sometimes report that they have the following clinical symptoms. Please indicate the extent to which you have experienced these symptoms or problems, please answer by circling the number that best applies to you.

During the past week:	Not at All	A Little	Quite a Bit	Very Much
31. Did you cough frequently?	1	2	3	4
32. Did you cough up blood (blood in sputum)?	1	2	3	4
33. Did you feel short of breath when you rest?	1	2	3	4
34. Did you feel short of breath when walking?	1	2	3	4
35. Did you feel short of breath when climbing the stairs?	1	2	3	4
36. Have you ever had pain in your mouth or tongue?	1	2	3	4
37. Have you ever had difficulty in swallowing?	1	2	3	4
38. Have you ever had numbness/tingling in your hands or feet?	1	2	3	4
39. Have you ever had hair loss?	1	2	3	4
40. Have you ever had chest pain?	1	2	3	4
41. Have you ever had pain in your arm or shoulder?	1	2	3	4
42. Have you had any pain in other parts of your body?	1	2	3	4
If yes, please write down the part of body:	1	2	3	4
43. Have you taken any analgetic?	1	2	3	4
1. No 2. Yes				
If yes, is it effective in relieving pain?	1	2	3	4

1. Calculation of Scores

EORTC's QLQ-C30 (V3.0) is a core scale for all cancer patients, with a total of 30 items. Among them, Item 29 and 30 are divided into seven grades, which are assigned with 1 to 7 scores according to the answer options. The other items are divided into 4 grades: Not at All, A Little, Quite a Bit, and Very Much, assigned with 1 to 4 scores respectively.

2. Calculation of Domain (Dimension) Score (Raw Score)

The scale is usually divided into several domains, for the convenience of statistical analysis and application. A domain is one of the components of life quality, also known as a "dimension", which is used as an independent variable in analysis.

The 30 items of E0RTC QLQ-C30 (V3.0) can be divided into 15 domains, including 5 functional domains (physical, role, cognitive, emotional and social functions), 3 symptomatic domains (fatigue, pain, nausea and vomiting), 1 general health status/life quality domain and 6 single items (each as a domain). The score of a domain (also referred to as "Raw Score") could be calculated by dividing sum of the scores of items included in the domain with the number of items, i.e. RS=(Q1+Q2+?Q)/n. (See Table 1.)

3. Calculation of Standard Score

The linear transformation could be made further by means of the range method to transform the raw score into a standard score (SS) within a range of 0-100, in order to conduct comparison between two domains. In addition, the transformation also has another purpose, i.e. to convert the direction of scoring. All the items in QLQ-C30 scale, except for items 29 and 30, are reverse items (the higher the value, the worse the quality of life). However, the scoring rules clearly stipulate that the higher scores of functional domains and general health status domain indicate the better functional conditions and quality of life, while the higher scores of symptomatic domains indicate the presence of more symptoms or problems (i.e. the worse quality of life). Therefore, the direction shall be converted when calculating the standard score of a functional domain. Specifically, it could be calculated by using the following formulas: (wherein R refers to the scoring difference between two domains or between two items).

Functional domain: $SS=[1-(RS-1)/R]\times 100$

Symptomatic domains and general health status domain: $SS=[(RS-1)/R]\times 100$

Physical function	(Q1+Q2+Q3+Q4+Q5)/5
Role function	(Q6+Q7)/2
Emotional function	(Q21+Q22+Q23+Q24)/4
Congnitive function	(Q20+ Q25)/2
Social function	(Q26+Q27)/2
Global QOL	(Q29+Q30)/2
Fatigue	(Q10+Q12+Q26)/3
Nausea and vomiting	(Q14+Q15)/2
Pain	(Q9+Q19)/2
Dyspnea	Q8
Sleep disturbance	Q11
Appetite loss	Q13
Constipation	Q16
Diarrhea	Q17
Financial difficulties	Q28
