# Supplementary material for: Neoadjuvant Afatinib for stage III EGFR-mutant non-small cell lung cancer: a phase II study
Source: Nat Commun. 2023 Aug 3;14:4655. doi: 10.1038/s41467-023-40349-z (PMC10400609; doi:10.1038/s41467-023-40349-z)
Supplement: Supplementary file 3 — Reporting Summary [file 41467_2023_40349_MOESM3_ESM.pdf]

nature portfolio

Corresponding author(s): Peng Zhang

Last updated by author(s): Jul 25, 2023

Reporting Summary

Nature Portfolio wishes to improve the reproducibility of the work that we publish. This form provides structure for consistency and transparency in reporting. For further information on Nature Portfolio policies, see our Editorial Policies and the Editorial Policy Checklist.

Statistics

For all statistical analyses, confirm that the following items are present in the figure legend, table legend, main text, or Methods section.

n/a	Confirmed	
☐	☒	The exact sample size (*n*) for each experimental group/condition, given as a discrete number and unit of measurement
☐	☒	A statement on whether measurements were taken from distinct samples or whether the same sample was measured repeatedly
☐	☒	The statistical test(s) used AND whether they are one- or two-sided<br>*Only common tests should be described solely by name; describe more complex techniques in the Methods section.*
☐	☒	A description of all covariates tested
☐	☒	A description of any assumptions or corrections, such as tests of normality and adjustment for multiple comparisons
☐	☒	A full description of the statistical parameters including central tendency (e.g. means) or other basic estimates (e.g. regression coefficient) AND variation (e.g. standard deviation) or associated estimates of uncertainty (e.g. confidence intervals)
☐	☒	For null hypothesis testing, the test statistic (e.g. *F*, *t*, *r*) with confidence intervals, effect sizes, degrees of freedom and *P* value noted<br>*Give P values as exact values whenever suitable.*
☒	☐	For Bayesian analysis, information on the choice of priors and Markov chain Monte Carlo settings
☒	☐	For hierarchical and complex designs, identification of the appropriate level for tests and full reporting of outcomes
☐	☒	Estimates of effect sizes (e.g. Cohen's *d*, Pearson's *r*), indicating how they were calculated

Our web collection on statistics for biologists contains articles on many of the points above.

Software and code

Policy information about availability of computer code

Data collection	Clinical data was recorded in CRF (case report form) in paper version, restored in Execl (version 16.73, Microsoft Excel for Mac)
Data analysis	Clinical data were analyzed by SPSS software (version 26.0, IBM Corp, Armonk, NY).<br>Exploratory analyses were conducted using R programing (version 4.1.0).<br>For RNAseq data, raw fastq files were trimmed via fastp (v0.20.1) and aligned to GRCh38 reference genome by STAR (v2.7.6a). After obtaining the BAM files, read counts were summarized by featureCounts (v2.0.1) and TPMs were generated using Salmon (v0.6.0). DESeq2 was used to calculate differential gene expression between sample groups. The immune scores of each sample were calculated using the "ESTIMATE" R package and the infiltration of multiple immune cells was evaluated by "mcpcounter" and "EPIC". TRUST4 algorithm was applied to evaluate the immune repertoire and to extract T and B cell receptor.

For manuscripts utilizing custom algorithms or software that are central to the research but not yet described in published literature, software must be made available to editors and reviewers. We strongly encourage code deposition in a community repository (e.g. GitHub). See the Nature Portfolio guidelines for submitting code & software for further information.

Data

Policy information about availability of data

All manuscripts must include a data availability statement. This statement should provide the following information, where applicable:

- Accession codes, unique identifiers, or web links for publicly available datasets
- A description of any restrictions on data availability
- For clinical datasets or third party data, please ensure that the statement adheres to our policy

The raw RNA-seq data generated in this study have been deposited in the Genome Sequence Archive of the BIG Data Center at the Beijing Institute of Genomics, Chinese Academy of Science, under accession code HRA003549 (accessible at https://ngdc.cncb.ac.cn/gsa-human/browse/HRA003549). The sequencing data are available under controlled access due to data privacy laws related to patient consent for data sharing and the data should be used for research purposes only. Access can be obtained by approval via the Data Access Committee in the GSA-human database. The approximate response time for accession requests is about 2 weeks. Once access has been granted, the data will be available to download for 3 months. Clinical data were not publicly available due to involving patient privacy, but can be accessed from the corresponding author Peng Zhang (Email: zhangpeng1121@tongji.edu.cn), upon request for 3 years; individual de-identified patient data will be shared for clinical study analyses. Data from two publicly available datasets were incorporated into our study: TCGA-LUAD level three RNA-seq data and clinical information from patients were acquired from the UCSC Xena website (https://xenabrowser.net/). Cell lines annotated as ''Non-Small Cell Lung Cancer (NSCLC), Adenocarcinoma'' with EGFR mutations from DepMap (https://depmap.org/) were applied with molecular profiles and drug response information used. Pearson correlation was conducted between CISH expression and drug responses information to Afatinib in PRISM secondary screen (https://depmap.org/portal/prism/) and CTRP database (https://portals.broadinstitute.org/ctrp).

Research involving human participants, their data, or biological material

Policy information about studies with human participants or human data. See also policy information about sex, gender (identity/presentation), and sexual orientation and race, ethnicity and racism.

Reporting on sex and gender	We declare that this clinical trial and following exploratory analysis do not involve sex and gender considerations for study involving participants according to the guidance of Nature journals.
Reporting on race, ethnicity, or other socially relevant groupings	This is a single-center trial conducted at Shanghai Pulmonary Hospital in China, involving participants who are exclusively Chinese and representing the East-Asian population. No subgroup analyses regarding race , ethnicity or other socially relevant groupings were performed.
Population characteristics	Patient data from a prospective clinical trial of Afatinib (the 2nd-generation EGFR-TKI) for stage III EGFR-mutant non-small cell lung cancer (NSCLC): TEAM-LungMate 004, which was registered with ClinicalTrial.gov (NCT04201756). Patients with stage III EGFR-mutant NSCLC enrolled in this trial were treatment-naive and harboring EGFR mutation.
Recruitment	Treatment-naive patients with stage III NSCLC with EGFR mutation were screened in Department of Thoracic Surgery, Shanghai Pulmonary Hospital, and recruited for study. The details of inclusion criteria of participants were shown in the registration website, as described above. This is a prospective, open-label, single-arm, phase II trial to assess the efficacy and safety of neoadjuvant Afatinib treatment. Besides, the experimental laboratory study on tumor tissues of participants to illustrate the prognostic biomarkers of target-therapy and the changes of tumor immune microenvironment after EGFR-TKI treatment. Informed consent was obtained from all human research subiects.
Ethics oversight	The study was approved by the independent ethic committee in Shanghai Pulmonary Hospital, Tongji University (19229XW).

Note that full information on the approval of the study protocol must also be provided in the manuscript.

Field-specific reporting

Please select the one below that is the best fit for your research. If you are not sure, read the appropriate sections before making your selection.

☒ Life sciences ☐ Behavioural & social sciences ☐ Ecological, evolutionary & environmental sciences

For a reference copy of the document with all sections, see nature.com/documents/nr-reporting-summary-flat.pdf

Life sciences study design

All studies must disclose on these points even when the disclosure is negative.

Sample size	The primary endpoint of this research was ORR, which was taken to calculate the sample size. Previous studies reported that the ORR of neoadjuvant Erlotinib for NSCLC (EMERGING-CTONG 1103) at stage III-N2 was 54.1% 12. The ORR in control group for neoadjuvant chemotherapy was 34.3%, and the ORR of neoadjuvant Afatinib for NSCLC (ASCENT) at stage III was 75%. Given that the small sample size of ASCENT trial (n=22) may lead to overestimate the ORR rate, and that Afatinib had better therapeutic effect as the generation-II TKI than the generation-I TKIs, so the ORR rate was estimated to be 60% in this study. The ORR rate was expected to be 60% in this study, and the neoadjuvant chemotherapy group of EMERGING trial was taken as the historical control group. It was calculated with α=0.05 (two-tailed) and the power of test (1-β) =90%. According to the sample calculation of one-sample rate test, when P0 =34%, and P1=60%, the sample size will be 42 as calculated. With a drop-off rate of 10%, the total sample size will be 47.

Data exclusions	Pregnant or breast-feeding patients, patients with unstable systemic disease (interstitial lung disease, pulmonary fibrosis, cardiovascular disease and so on), patients with any anticancer therapy outsides of this trial (EGFR-TKIs, chemotherapies, immunotherapies, and so on), and patients with exon 20 codon p.Thr790Met point mutation (T790M) or exon 20 insertion mutation (Ex20Ins) were ineligible for this study.
Replication	Bulk-RNA-seq and IHC-score data were not applicable for replication due to inability to acquire multiple fresh specimens from the same participants. However, the transcriptional signatures identified in our dataset were consistent with prior studies.
Randomization	None (single-arm study).
Blinding	None (single-arm study).

Reporting for specific materials, systems and methods

We require information from authors about some types of materials, experimental systems and methods used in many studies. Here, indicate whether each material, system or method listed is relevant to your study. If you are not sure if a list item applies to your research, read the appropriate section before selecting a response.

Materials & experimental systems

n/a	Involved in the study	
☐	☒	Antibodies
☒	☐	Eukaryotic cell lines
☒	☐	Palaeontology and archaeology
☒	☐	Animals and other organisms
☐	☒	Clinical data
☐	☒	Dual use research of concern
☒	☐	Plants

Methods

n/a	Involved in the study	
☒	☐	ChIP-seq
☒	☐	Flow cytometry
☒	☐	MRI-based neuroimaging

Antibodies

Antibodies used	Anti-CISH/CIS antibodv-C-terminal (Abcam: ab191447):<br>Anti-PD-L1 antibody (PD-L1: E1L3, Rabbit mAb, CST: 13684S).
Validation	All the antibodies used in this study were commercial available antibodies, with validation procedures described on the manufacturer's websites as below:<br>Anti-CISH/CIS antibody-C-terminal (Abcam: ab191447) (https://www.abcam.cn/products/primary-antibodies/cishcis-antibody-c-terminal-ab191447.html#description protocols; validated in human for IHC)<br>Anti-PD-L1 antibody (PD-L1: E1L3, Rabbit mAb, CST: 13684S) (https://www.abcam.cn/products/primary-antibodies/pd-11-antibodv-73-10-low-endotoxin-azide-free-ab246698html: validated in human for IHC)

Clinical data

Policy information about clinical studies

All manuscripts should comply with the ICMJEguidelines for publication of clinical research and a completedCONSORT checklist must be included with all submissions.

Clinical trial registration	NCT04201756, https://clinicaltrials.gov/ct2/show/NCT04201756
Study protocol	These are a phase II, non-randomized, open-label, studies to evaluate the efficacy of neoadjuvant Afatinib as first-line treatment in patients with stage III non-small cell lung cancer harboring EGFR mutation (NSCLCm+). The participants receive Afatinib, 40mg, qd, oral, 2-4 cycles; then receive surgery. The aim of the study attempted to access the safety and efficacy of neoadjuvant Afatinib monotherapy for stage III NSCLCm+. Besides, exploratory analysis attempted to delineate the dynamics of the tumor microenvironment during target-therapy and explore the potential mechanisms behind treatment resistance. In this study, bulk-RNA-seq and IHC-score were applied in the primary tumor specimens before and after Afatinib treatment. The full trial protocol could be viewed on website (clinicaltrials.gov), and will also be uploaded to Nature protfolio in the Supplementary Files.
Data collection	The clinical data was record in the Case Report Form (CRF) in paper version and was stored in Excel. Besides, we collected the primary tumor tissue by percutaneous pulmonary biopsy, bronchoscopy biopsy or endobronchial ultrasound (EBUS) biopsy before and the tumor tissue by surgery before and after drug administration respectively. The fresh tumor tissues were collected immediately after biopsy and surgical resection for RNA-seq. Tumor samples were fixed with formalin and embedded in paraffin for IHC.<br>The detailed clinical information of each participant was collected in the GCP Drug Clinical Research Office of our center at the specified time point according to the GCP drug clinical research specifications.
Outcomes	The primary endpoint was objective response rate (ORR) accessed by the RECIST criteria. As it stated in manuscript, previous studies reported that the ORR of neoadjuvant Erlotinib for NSCLC (EMERGING-CTONG 1103) at stage III-N2 was 54.1%, Afatinib had better therapeutic effect as the generation-II TKI than the generation-I TKIs, so the ORR rate was estimated to be 60% in this study.The trial successfully achieves the pre-specified primary endpoint that the objective response rate (ORR) reaches 70.2%.<br>The secondary endpoints were MPR rate (proportion of patients with no more than 10% residual viable tumor cells), the complete resection rate, EFS, OS, and TRAEs. TRAEs were recorded in CRF (case report form). MPR rate and complete resection rate were calculated after all 33 patients received neoadjuvant treatment followed by surgery. With the survival data maturity of follow-up, the EFS and OS of this trial could be reported.
